# Selective targeting and clustering of phosphatidylserine lipids by RSV M protein is critical for virus particle production

**DOI:** 10.1016/j.jbc.2023.105323

**Published:** 2023-10-05

**Authors:** Jitendriya Swain, Maxime Bierre, Laura Veyrié, Charles-Adrien Richard, Jean-Francois Eleouet, Delphine Muriaux, Monika Bajorek

**Affiliations:** 1 Virology and Molecular Immunology Unit (VIM), Animal Health Department, INRAE, IRIM, Montpellier, France; 2 INRAE, UVSQ, VIM, Université Paris-Saclay, Jouy-en-Josas, France

**Keywords:** lipid-protein interaction, lipid bilayer, RNA virus, membrane lipid, phosphatidylserine, RSV matrix protein, M phosphorylation, LUVs

## Abstract

Human respiratory syncytial virus (RSV) is the leading cause of infantile bronchiolitis in the developed world and of childhood deaths in resource-poor settings. The elderly and the immunosuppressed are also affected. It is a major unmet target for vaccines and antiviral drugs. RSV assembles and buds from the host cell plasma membrane by forming infectious viral particles which are mostly filamentous. A key interaction during RSV assembly is the interaction of the matrix (M) protein with cell plasma membrane lipids forming a layer at assembly sites. Although the structure of RSV M protein dimer is known, it is unclear how the viral M proteins interact with cell membrane lipids, and with which one, to promote viral assembly. Here, we demonstrate that M proteins are able to cluster at the plasma membrane by selectively binding with phosphatidylserine (PS). Our *in vitro* studies suggest that M binds PS lipid as a dimer and upon M oligomerization, PS clustering is observed. In contrast, the presence of other negatively charged lipids like PI(4, 5)P2 does not enhance M binding beyond control zwitterionic lipids, while cholesterol negatively affects M interaction with membrane lipids. Moreover, we show that the initial binding of the RSV M protein with PS lipids is independent of the cytoplasmic tail of the fusion (F) glycoprotein (FCT). Here, we highlight that M binding on membranes occurs directly through PS lipids, this interaction is electrostatic in nature, and M oligomerization generates PS clusters.

Respiratory syncytial virus (RSV) is a major public health issue. Human RSV is the most frequent cause of infantile bronchiolitis and pneumonia worldwide. In France, 460,000 infants are infected each year, and it is the first cause of hospitalization of young children. RSV hospitalization in elderly is comparable to influenza. The enormous burden of RSV makes it a major unmet target for a vaccine and antiviral drug therapy (1). Recently, RSV vaccines for adults (Pfizer, GSK) and an mAb for infants were announced (AstraZeneca/Sanofi), but both miss important targets like infant vaccination and affordable therapies for low-income countries. The lack of knowledge of the RSV assembly and budding mechanism also presents a continuing challenge for large scale virus-like particle (VLPs) production for vaccine purposes. Therefore, understanding RSV assembly mechanism could potentially open a new platform for both therapeutic strategy and vaccine development.

RSV belongs to the *Pneumoviridae* family in the order *Mononegavirales* (2). According to the common paradigm, RSV assembles on the plasma membrane, and infectious viral particles are mainly filamentous (3, 4). However, more recent data suggest that viral filaments are produced and loaded with genomic RNA prior to insertion into the plasma membrane. According to this model, vesicles with RSV glycoproteins recycle from the plasma membrane and merge with intracellular vesicles, called assembly granules, containing the RNPs (5, 6). RSV virions then assemble and bud (7) forming infectious viral particles which are mainly filamentous (8). The minimal set of RSV proteins required for viral filament formation are the cytoplasmic tail of fusion (F) glycoprotein (FCT), the phosphoprotein (P), and the matrix (M) protein (9, 10). M, a key structural protein, directs assembly, forming a protein lattice at specific assembly sites underlaying the plasma membrane (8, 11, 12, 13, 14). Earlier studies using cryo-EM of culture-grown RSV have determined the architecture of the virus. The presence of an intact M layer beneath the viral membrane was linked to the virion's prefusion F form (8). Most recent cryo-electron tomography data has shown a helical lattice of M organized as dimers beneath the viral membrane, further confirming that M has implications on the conformation of the F protein (14). These M lattices were suggested to bridge viral glycoproteins *via* the FCT and the internal RNP complex, the latter *via* binding to oligomeric N and/or P associated with the viral genome (10, 13). Moreover, FCT was shown to be required for infectious virus production. FCT was shown to be essential for viral filament formation (15) and for budding, specifically the three last amino acids (F22-S23-N24) of the tail (16). Recently, M and P alone were shown to form a sufficient platform for VLP budding, producing particles that were highly variable in shape and size (17), suggesting that F is required for filament formation but not for the budding itself.

M is a main driver of RSV filament formation and budding (9, 12, 18). Previous structural data from our group showed that M forms dimers that are critical for viral filament assembly and VLP production (19). RSV M unstable dimers result in defects in higher-order oligomerization and as a consequence lack of filament formation and budding (19, 20). Additionally, M carrying phosphomimetic residue substitution that causes extensive higher-order oligomer assembly and aggregation leads to uninfectious virus production (4).

The lipidic envelope of RSV, and many other enveloped viruses, such as HIV-1 (21), comes from the host cell plasma membrane. The plasma membrane bilayer consists of an outer leaflet and an inner leaflet. The major lipid of the outer leaflet is phosphatidylcholine (PC) (ranges from 76–78%), and others lipids like sphingomyelin (SM) and glycosphingolipids are present in lesser quantities. The inner leaflet is enriched with phosphatidylethanolamine (PE) (ranges from 0-73 %), phosphatidylserine (PS) (ranges from 0–40%), and lesser amount of phosphoinositides (PI(4,5)P2) (0–5%) and phosphatidylglycerol (PG) (0–4%) (22, 23). PS is the most abundant anionic lipid (net negative charge −1) in the inner leaflet of the plasma membrane and plays a major role in the recruitment of a number of viral matrix proteins for assembly and budding processes (24). Specifically, matrix of filoviruses, VP40, Ebola virus (EBOV) and Marburg virus (MARV) selectively binds membrane that contain PS, while PI(4,5)P2 stabilize VP40 oligomerization and thus positively affects budding (25, 26, 27, 28). Our previous works on retroviruses reveal that retroviral Gag hijack PI(4,5)P2, and sometimes PS, creating a platform for assembly, independently of envelope glycoproteins but strongly dependent on Matrix residues (29, 30, 31, 32). Clustering of PI(4,5)P2 lipids enriched in cholesterol but not in PE or SM was demonstrated during HIV-1 assembly (32). This was in contrast with the influenza matrix protein, M1, which showed interaction only with PS-enriched membranes with no contribution of PI(4,5)P2 (33). Different viruses can also affect membrane lipid biogenesis. Some examples include the Picornavirus 3CD protein that induces PI4P, PIP2, and PC synthesis (34) or the Vaccinia virus H7 protein that binds PI3P and PI4P and regulates membrane lipid biogenesis (35).

The interaction of RSV M with specific lipids during assembly is not fully understood. The sorting of RSV M protein into plasma membrane lipid rafts has been well documented and found to be dependent on the presence of cell surface glycoproteins. However, in their absence, M protein is still present at the plasma membrane but not concentrated in lipid rafts (36), suggesting selective M binding to certain lipids prior to budding. Each monomer of M protein comprises two compact β-rich domains connected by an unstructured linker region. An extensive contiguous area of positive surface charge covering 600 Å^2^ and spanning both domains was previously suggested to drive the interaction with negatively charged membrane surface. This positive region is complemented by regions of high hydrophobicity and a striking planar arrangement of tyrosine residues encircling the C-terminal domain, which make it suitable to target different phospholipids of plasma membrane surface (37). *In vitro* studies have shown that RSV M protein interacts with lipid monolayers with neutral lipid compositions (DOPC/DPPC/cholesterol and DOPC/SM/cholesterol), and this interaction does not appear to be affected by the hydrophobic effect or the presence of cholesterol (38). To our knowledge, a systematic study of M interaction with specific plasma membrane lipids has not been yet conducted.

In this work, we demonstrate that the interaction between RSV M protein and the host cell membrane is selectively facilitated by the PS lipid. Our findings show that the binding of M to lipids is not dependent on the presence of other highly negatively charged lipids like PI(4, 5)P2 or PG and that hydrophobic cholesterol negatively impacts initial M binding. Our results indicate that M alone can induce clustering of PS lipids, and the interaction between M protein and lipids does not require the FCT protein. Our study, based on M mutant proteins *in vitro*, also suggests that M binds PS lipids as dimers. M mutant with an extensive oligomerization inhibits interaction with PS lipids, whereas lack of ordered M oligomerization negatively affects PS clustering. Furthermore, we confirm that M, P, and FCT are all necessary for the formation of RSV-like filaments, as reported (9, 10), but M and P are the minimum components required to form VLPs. This is, to our knowledge, the first demonstration of M interaction with physiologically relevant PS-enriched membrane lipids *in vitro* and can be used to further investigate the complexity of RSV assembly of budding mechanisms.

## Results

### M selectively interacts with negatively charged PS lipid in the presence of neutral and anionic lipid

**Figure 1:**
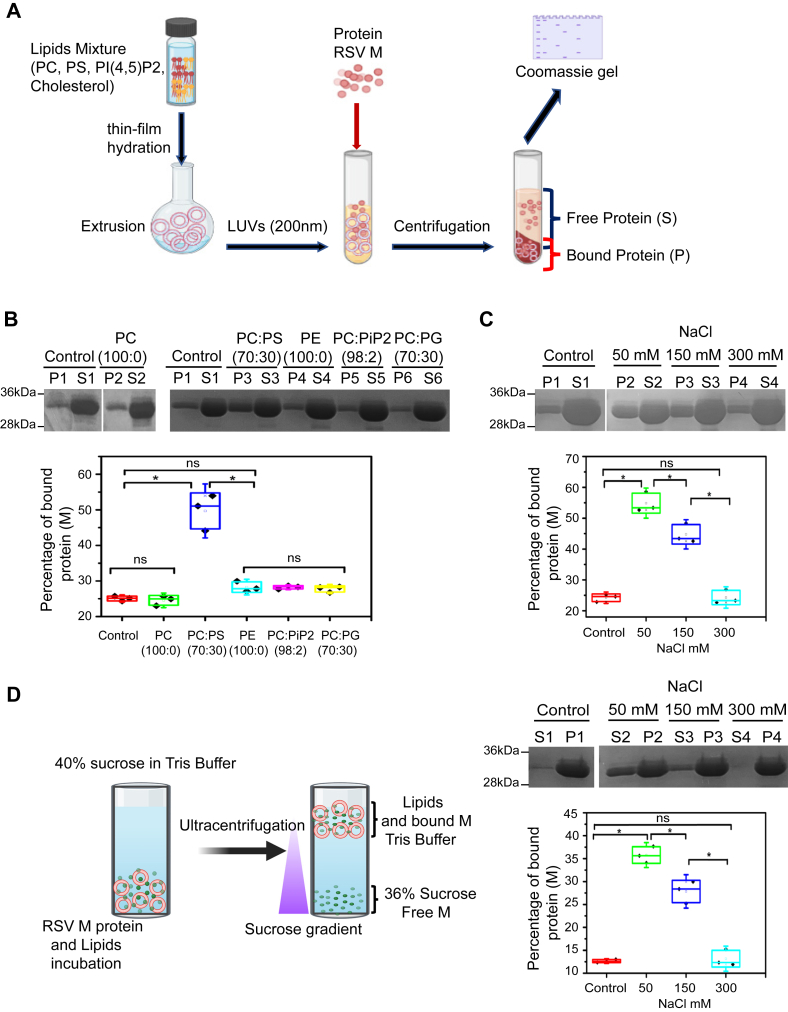
**M interacts selectively with negatively charged phosphatidyl-Serine and with electrostatic interactions.***A*, schematic representation of protein-LUV cosedimentation assay used. *B*, representative SDS-PAGE obtained after LUV cosedimentation assay and staining with Coomassie blue showing RSV M protein (15 μM) negative control (P1,S1) (in the absence of lipids) and with PC (100:0) (P2,S2), PC:PS (70:30) (P3,S3), PE (100:0) (P4,S4), PC: PI(4,5)P2 (98:2) (P5,S5), or PC: PG (70:30) (P6,S6) LUVs (pellet (P) and supernatant (S)). The percentage of bound RSV M protein to lipids (P) was quantified and is shown as a graph below. *C*, representative SDS-PAGE obtained after cosedimentation assay and staining with Coomassie blue showing RSV M and PC:PS (70:30) lipids binding, control (P1,S1) (in the absence of lipids), and with NaCl 50 mM (P2,S2), NaCl 150 mM (P3,S3), or NaCl 300 mM (P4,S4). The percentage of bound RSV M protein to lipids (P) at different NaCl concentrations was quantified and is shown as a graph below. *D*, schematic representation of the LUV-flotation assay used in this study as shown on the *left*. Representative SDS-PAGE obtained after the LUV-flotation assay and staining with Coomassie blue showing RSV M and PC:PS (70:30) lipids binding, control (S1,P1) (in the absence of lipids), and with NaCl 50 mM (S2,P2), NaCl 150 mM (S3,P3), or NaCl 300 mM (S4,P4). Here, P is showing the unbound M and S is showing the LUV-bound proteins. The percentage of bound M protein to LUVs (S) at different NaCl concentrations was quantified and is shown as a graph below. For graphs in *B*, *C*, and *D*, each point is a mean ± SD of n = 3 independent experiments. One-way ANOVA and Mann-Whitney test was used for group comparisons. ns, *p* > 0.05, ∗*p* ≤ 0.05. LUV, large unilamellar vesicle; PC, phosphatidylcholine; PE, phosphatidylethanolamine; PG, phosphatidylglycerol; PS, phosphatidylserine; RSV, respiratory syncytial virus.

Although it is known that RSV M binds inner leaflet of plasma membrane lipids during assembly, it is unclear whether the interaction is nonspecific towards negatively charged membrane surface or rather particular lipid head groups anchor M to the inner membrane leaflet. To study RSV M interaction with lipids, we first used liposome sedimentation assays and large unilamellar vesicles (LUVs) (Fig. 1*A*) with different lipid composition to identify specific lipids critical for interaction under physiological salt concentration. The physiological concentration of monovalent salt ions (primarily sodium, potassium, and chloride) in the human body is on the order of 150 mM, and potassium is predominant compared to sodium. We choose to use 150 mM NaCl at pH (7.4) in our experiments (Fig. 1*B*) and 200 nm LUVs for this study (Fig. S1). LUVs with PC (100:0), PC:PS at molar ratio of 70:30, PE (100:0), PC: PI(4,5)P2 (98:2), and PC:PG at molar ratio 70:30 were prepared and incubated with M protein as described in the experimental procedure section. M incubation without LUVs served as negative control. Samples were then centrifugated to separate the supernatant (S) fraction containing the unbound soluble M from the pellet (P) fraction containing the LUV-bound M. Both fractions were migrated on SDS gel and stained with Coomassie blue for M visualization and quantification by densitometry. As shown in Figure 1*B*, about 25% of M was found in the P fraction in the absence of LUVs. This agrees with previous results showing some RSV M oligomerization and precipitation over time (4, 19). This is also the most likely reason for variable percentage of RSV M without lipids found in the P fraction in the different sets of experiments. Thus, it appears that the M oligomerization state is concentration- and time-dependent and it varies among the different sets of protein preparation. In the presence of the most predominant PC lipids, up to 24.5% of M was identified in the LUV-bound fraction (P). This is comparable to M in the P fraction in the absence of lipids, indicating no binding. In contrast, M protein bound up to 49.7% to LUVs with PC:PS (70:30) lipid mixtures, which was significantly higher than negative control (Fig. 1*B*). Incubation of M with LUVs with other lipid combinations, such as PE (100:0), PC:PI(4,5)P2 (98:2), or PC:PG (70:30), resulted in M proteins in the P fraction at a percentage equivalent to the negative control, that is, up to 28.3%, 28.2%, and 27.7%, respectively (Fig. 1*B*). Our findings demonstrate that, in the presence of LUVs enriched in different lipids, RSV M selectively interacts with negatively charged PS lipids. Further, to show the electrostatic nature of the interaction between the positive surface of RSV M and the negatively charged PS lipids, we performed the same sedimentation assay with PC:PS (70:30) LUVs in the presence of different NaCl concentrations (50 mM, 150 mM, 300 mM). As shown in Figure 1*C*, increased salt concentration resulted in reduced percentage of LUV-bound M proteins, ranging from 54.8% in 50 mM to 24.3% in 300 mM salt, confirming the electrostatic nature of this interaction. Next, to verify that our results in Figure 1, *B* and *C* were not due to M sedimentation only, we used lipid flotation assays to confirm M–lipid interactions (Fig. 1*D*). In this assay, M protein is incubated with LUVs and loaded at the bottom of the tube followed by a centrifugation through a sucrose gradient. Only M protein bound to LUVs will float (S fractions), and unbound M protein will remain in the pellet (P fractions). PC:PS (70:30) LUVs were prepared and incubated with M protein in the presence of different NaCl concentration (50 mM, 150 mM, 300 mM). M protein incubation without LUVs served as negative control. Both fractions were migrated on SDS gel and stained with Coomassie blue for M visualization and quantification. As shown in Figure 1*D*, M protein in the absence of LUVs (which in this experiment includes both the soluble and the oligomeric protein) was mostly found in the P fraction, while only 12.6% of M was found in the S fraction. In contrast, when M was incubated with PC:PS LUVs in the presence of 50 mM, 150 mM, and 300 mM NaCl, LUV-bound M consisted of 35.8%, 27.8%, and 13.2%, respectively. The percentage of LUV-bound M thus decreased with increased salt concentration, which confirmed our sedimentation assay results (Fig. 1*C*). Our results in Figure 1, using two complementary assays, show that RSV M selectively interacts with PS lipids and that this interaction is electrostatic in nature.

### Quantification of RSV M binding to PS lipids using LUV cosedimentation assays

**Figure 2:**
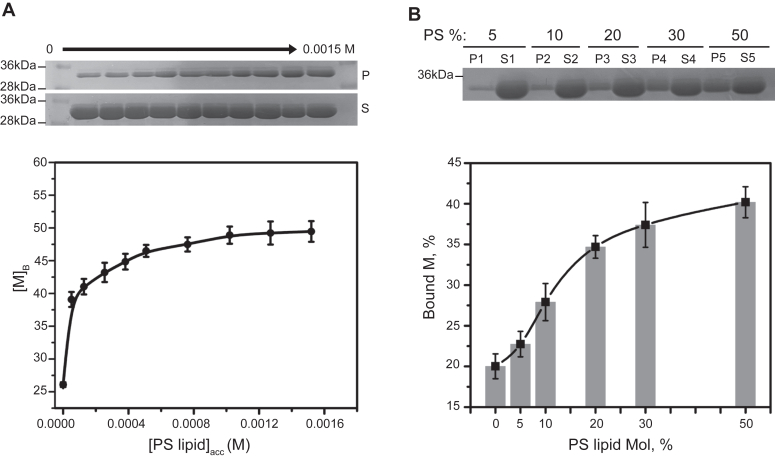
**Quantification of RSV M binding affinity to PS lipid using LUV cosedimentation assays.***A*, SDS-PAGE obtained after cosedimentation assay and staining with Coomassie blue for RSV M protein visualization and quantification. Gel image corresponding to the binding of M (8 μM) to PC:PS (70:30) LUVs (from *left* to *right*, with increasing accessible lipid concentration from 50 μM to 1.5 mM); P corresponds to the pellet of M protein bound to LUV and S to the supernatant of unbound M. The plot shows the percentage of M bound ([M]_B_) (y -axis) with accessible PS lipid concentrations in mol ([PS lipid]acc (x-axis)). Each point is a mean of n = 3 independent experiments with SD. *B*, binding of M to LUVs with increasing mol percentage of PS concentrations. Binding of M (8 μM) to 1 mg/ml LUVs composed of 0 to 50 mol percent of PS lipid. P corresponds to the pellet (LUV-bound M) and S to the supernatant (unbound M). The percent of bound M protein is plotted (bar plot) as a function of the mol percentage of PS in the LUVs. The curve is fitted with the Hill equation (Eq. 4) (see Experimental procedures). Each point is mean ± SD of n = 3 independent experiments. LUV, large unilamellar vesicle; PC, phosphatidylcholine; PS, phosphatidylserine; RSV, respiratory syncytial virus.

Further, we tried to calculate an apparent binding constant (*K*_d_, app) for the interaction between RSV M (at fixed concentrations of 8 μM) and LUVs of PC:PS (70:30) (mol%) composition with varying accessible PS phospholipid content (Fig. 2*A*). Same quantitative cosedimentation assays were used as in Figure 1. Figure 2*A* shows gel representation of M in P and S fractions with increased concentration of available PS lipids (from 50 μM to 1.5 mM) in LUVs. When the amount of accessible PS phospholipid in LUVs increased, M proteins were more likely to bind to PS lipids.

After trying different experimental fitting of the raw data (see Experimental procedures), the apparent *K*_d,app_ was estimated between 15 and 57 μM (Figs. 2*A* and S2). This value cannot be more precise due to the limitation of the LUV sedimentation assay and would require other biophysical assays to access more precise *K*_d_.

It is well known that proteins can interact with multiple lipids in the membrane to increase protein-membrane affinity (39). We thus wanted to check whether RSV M binding to PS containing LUVs was cooperative. The assay for probing this question differed from the one in Figure 2*A*. Here, the total mol % of PS lipid concentration was varied (Fig. 2*B*). RSV M-lipid binding increased with rising PS mol % (5–50 mol %) and a sigmoidal binding curve was observed (Fig. 2*B*). Therefore, the sigmoidal dependence of M-lipid binding on the percentage of PS was fitted using the Hill equation (Eq. 4), which gave an n = 1.62. Thus, the binding stoichiometry being superior to 1, the interaction of RSV M with PS appears to be cooperative in nature, suggesting that oligomerization of M on PS membranes can be enhanced by M membrane binding.

### Other anionic lipids or cholesterol do not increase M binding to PS-enriched LUVs

**Figure 3:**
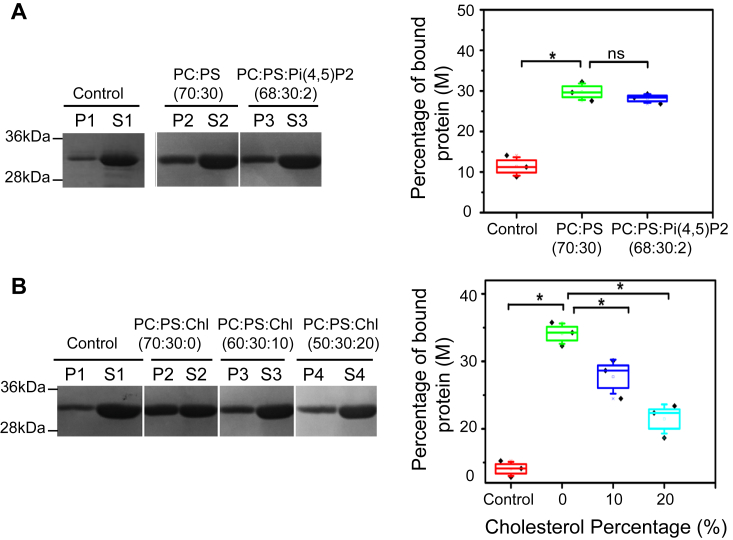
**M interaction with PS in the presence of PI(4, 5)P2 or cholesterol.***A*, SDS-PAGE obtained after cosedimentation assay and stained with Coomassie blue showing RSV M protein negative control (P1,S1) (in the absence of lipids), PC:PS (70:30) (P2,S2), and PC:PS:PI(4,5)P2 (68:30:2) (P3,S3) lipid (pellet (P) and supernatant (S)). The percentage of bound RSV M protein to LUVs (P) was quantified and is shown as a graph. *B*, SDS-PAGE obtained after M-LUV cosedimentation assay and stained with Coomassie blue showing RSV M proteins binding with different percentage of cholesterol (Chl), negative control (P1,S1) (in the absence of LUVs), M with PC:PS:Chl (70:30:0) (P2,S2), PC:PS:Chl (60:30:10) (P3,S3), and PC:PS:Chl (50:30:20) (P4,S4) lipid (pellet (P) and supernatant (S)). The percentage of bound RSV M protein to LUVs (P) was quantified and is shown as a graph. Each point is mean ± SD of n = 3 independent experiments. One-way ANOVA and Mann-Whitney test was used for group comparisons. ns, *p* > 0.05, ∗*p* ≤ 0.05 as indicated. LUV, large unilamellar vesicle; PC, phosphatidylcholine; PS, phosphatidylserine; RSV, respiratory syncytial virus.

Recently, it was reported that paramyxovirus Nipah and measles M proteins interact with negatively charged PS lipids and that PI(4,5)P2 significantly enhances this interaction (28). To examine the effect of PI(4,5)P2 on RSV M protein interaction with PS-LUVs, we conducted liposome sedimentation assays using PC:PS LUVs with and without PI(4,5)P2 (Fig. 3*A*). The results show that when incubated with PC:PS LUVs, M percentage increased in the P fraction (29.8%), reflecting LUV-bound M, compared to the negative control without lipids (11.3%). Similarly, when incubated with PC:PS:PI(4,5)P2 LUVs, the percentage of M in the P fraction (28.1%) increased 2.5 times compared to the negative control (Fig. 3*A*). This suggests that the presence of PI(4,5)P2 does not increase RSV M binding ability to PC:PS LUVs.

The role of lipid rafts and Caveolae hydrophobicity is well established in RSV assembly and budding (36, 40, 41), but the specific role of cholesterol for M binding to lipids has not been investigated. To study the effect of cholesterol on RSV M binding to lipids, we conducted sedimentation assays using PC:PS LUVs with and without different percentages of cholesterol (Fig. 3*B*). When M was incubated with PC:PS LUVs, 34.1% of M was found in the P fraction, which was significantly higher than the negative control without LUVs (14.0%). However, incubation with PC:PS:Chl (60:30:10) and PC:PS:Chl (50:30:20) LUVs resulted in 27.7% and 21.4% of M in the pellet, respectively. These results show that cholesterol significantly reduces the binding ability of RSV M to PC:PS LUVs.

### The FCT protein blocks RSV M protein-PS lipid binding on LUVs

**Figure 4:**
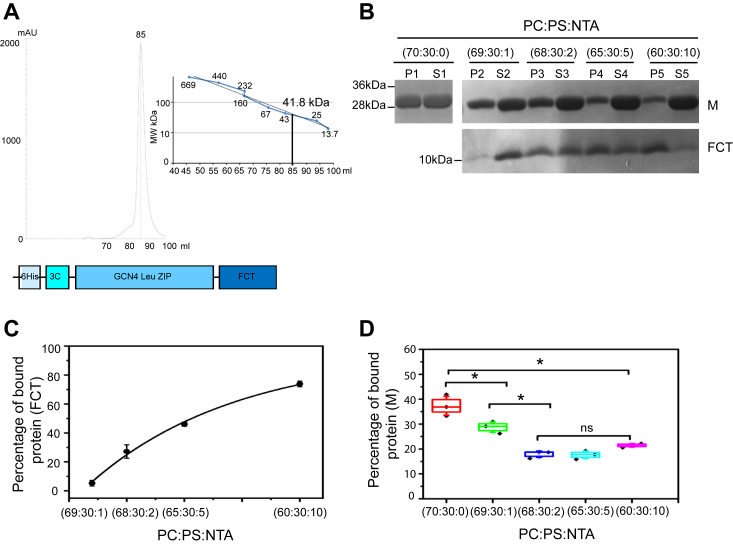
**The cytoplasmic tail of RSV F protein (FCT) blocks RSV M protein-PS lipid binding on LUVs.***A*, analytical size-exclusion chromatography of His_6_-GCN4-FCT. Molecular mass of the FCT protein was estimated at 41.8 kDa by comparing the gel-phase distribution of the FCT peak with the values obtained for known calibration protein standards on HiLoad 10/600 Superdex S200 column (GE Healthcare) and is marked on the curve (y = 29087e^–0.077x^). The schematic description of the His_6_-GCN4-FCT construct is shown below. *B*, SDS-PAGE obtained after LUV cosedimentation assay and staining with Coomassie blue showing RSV M and His_6_-GCN4-FCT protein binding to PC:PS:NTA (70:30:0) (P1,S1), PC:PS:NTA (69:30:1) (P2,S2), PC:PS:NTA (68.5:30:2.5) (P3,S3), PC:PS:NTA (65:30:5) (P4,S4), and PC:PS:NTA (60:30:10) (P5,S5) LUVs (pellet (P) and supernatant (S) are indicated). *C*, a graph showing the percentage of bound His_6_-GCN4-FCT protein incorporated into LUVs. *D*, a graph showing the percentage of bound RSV M to LUVs in the presence of different NTA concentrations. Each point is the mean ± SD of n = 3 independent experiments. One-way ANOVA and Mann-Whitney test was used for group comparisons. ns, *p* > 0.05, ∗*p* ≤ 0.05. LUV, large unilamellar vesicle; PC, phosphatidylcholine; PS, phosphatidylserine; RSV, respiratory syncytial virus.

The RSV FCT protein has been shown to be crucial for the production of filamentous and infectious virus (16, 42). A loss of interaction with cellular partners or other RSV proteins, M or P, has been suggested as a reason for the lack of virus budding in the presence of FCT mutations (16). The F protein is known to be a trimer (43), therefore resulting in three FCTs in the cytoplasm, which are believed to be disordered. In order to produce a trimeric FCT, we used a GCN4-mutated leucine zipper domain (MKQIEDKIEEILSKIYHIENEIARIKKLIGE) which is known to induce trimerization (44) and is one of the most widely used trimerization domains in research (45). A construct of Histidine tag and GCN4 leucine zipper domain fused to RSV FCT was made (Fig. 4*A*) to mimic FCT natural state. The trimeric His-GCN4-FCT protein was purified as described in experimental procedures. The purified protein migrated on SDS-PAGE according to its monomeric size of 9kD with an additional weak band migrating at 27kD and detected only by Western blotting using an anti-His antibody, most probably corresponding to a trimer (Fig. S3). The sizing column profile clearly showed that His-GCN4-FCT migrated similarly to 41.8kD protein (Fig. 4*A*), reflecting its oligomeric state. The slightly higher, 41.8kD instead of 27kD, estimated mass of a trimeric His-GCN4-FCT (monomer correspond to 9kD) probably reflects the unstructured nature of FCT domain. Unstructured proteins usually result in higher-than-expected protein migration on sizing column. To study the direct interaction between RSV M and FCT, different concentrations of 1,2-dioleoyl-sn-glycero-3-[(N-(5-amino-1-carboxypentyl)iminodiacetic acid)succinyl] (Cobalt salt) (DGS) NTA-Ni lipid were added to PC:PS lipid mixtures to bind the His-GCN4-FCT protein to LUVs, showing an increase in His-GCN4-FCT protein binding (up to 73.85%) with an increase in the concentration of DGS Ni-lipid (Fig. 4*B*, lower panel and 4C). The results from a sedimentation assay using His-GCN4-FCT-LUVs and M showed that increasing FCT concentration significantly reduces the binding ability of M protein to PC:PS LUVs, decreasing from a mean of 37.34% to about 17.2% (Fig. 4*B*, upper panel and *D*). This suggests that RSV M needs to directly interact with PS lipids and increasing concentration of FCT blocks M access to PS lipids.

### RSV M protein induces clustering of PS lipid on model membranes *in vitro*

**Figure 5:**
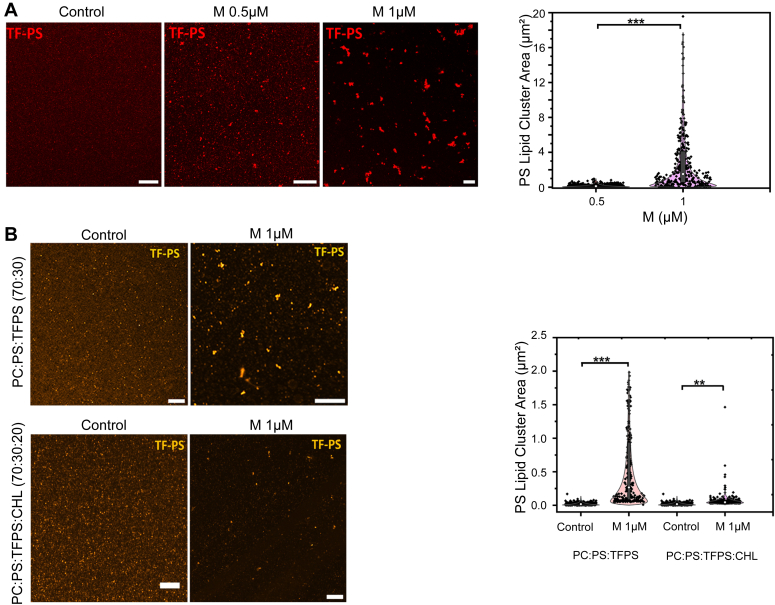
**RSV M protein induces clustering of PS lipid.***A*, confocal images of supported lipid bilayer (SLB) containing 70% PC and 30% PS lipids, with a fluorescent PS lipid (*Top* Fluor-PS) with and without RSV M protein (0.5 μM and 1 μM) (following incubation of 30 min, number of clusters N = 515, 315 for 0.5 μM and 1 μM, respectively). The plot shows PS clusters size in the presence of RSV M protein. *B*, high resolution Airyscan images of SLBs containing 70% PC and 30% PS lipids or 70% PC, 30% PS lipids, and 20% cholesterol, with a fluorescent PS lipid (*Top* Fluor-PS), in the absence or presence of M (1 μM). The plot shows quantification of PS lipid clusters size (number of clusters N = 350, 340, 201, and 250 for SLBs control without M WT, control without M in the presence of cholesterol, with M WT, and with M WT in the presence of cholesterol, respectively). All scale bars represent 5 μm. Each point is a cluster, n = 3 independent experiments. One-way ANOVA and Mann–Whitney test was used for group comparisons. ns, *p* > 0.05, ∗*p* ≤ 0.05, ∗∗*p* ≤ 0.01, ∗∗∗*p* ≤ 0.001. PC, phosphatidylcholine; PS, phosphatidylserine; RSV, respiratory syncytial virus.

Our findings above demonstrate that RSV M binds to PS lipids with strong selectivity and does not require the presence of other lipids or cholesterol (Figure 1, Figure 2, Figure 3, Figure 4). Here, to our knowledge for the first time, we examined the clustering ability of M towards PS lipids on model membranes *in vitro*. To do this, we used a supported lipid bilayer (SLB) containing 70% PC and 30% PS lipids, with a fluorescent PS lipid (Top Fluor-PS, 0.2 mol %). Using confocal imaging, we observed that RSV M quickly induced PS clusters on the SLBs. Figure 5*A* shows the clustering of PS lipids with and without M proteins. There were no or very few PS clusters in the absence of M (negative control). In contrast, adding 0.5 μM or 1 μM of M induced PS cluster formation. We also measured PS clusters size and found that they increased in a concentration-dependent manner (Fig. 5*A*), from 0.16 μm^2^ to 2.86 μm^2^, with 0.5 μM and 1 μM of M respectively (after 30 min of incubation). Overall, our results show that RSV M is able to cluster PS on model membranes, most probably a signature of RSV M oligomerization and assembly on membranes.

In addition, we used the same system of SLB containing 70% PC and 30% PS lipids, along with a fluorescent PS lipid (Top Fluor-PS, 0.02%) to measure PS clusters size at higher lateral optical resolution (140–150 nm using AIRY scan confocal microscopy) (Fig. 5*B*). In order to evaluate how cholesterol could influence the behavior of M clustering toward PS lipids on SLBs, we also explored 20% cholesterol in the presence of PC (50%) and PS (30%) lipids. There were no or very few PS clusters in the absence of M (negative control); this was regardless of if cholesterol was present or absent (compare upper and lower panels of the negative controls). We observed that when comparing the PS clusters size in the absence and in the presence of M, it significantly increased from 0.02 μm^2^ to 0.54 μm^2^. Additionally, the PS cluster size is substantially smaller in the presence of cholesterol (20%) and drops to 0.06 μm^2^ in comparison to in the presence of M and in the absence of cholesterol. This means that the capacity of RSV M protein to form PS clusters is greatly reduced by cholesterol. These results correlate with those obtained by LUV cosedimentation assays (Fig. 3*B*).

### RSV M binds PS lipids as dimer and M-controlled oligomerization is associated with PS clustering and virus-like filament formation

**Figure 6:**
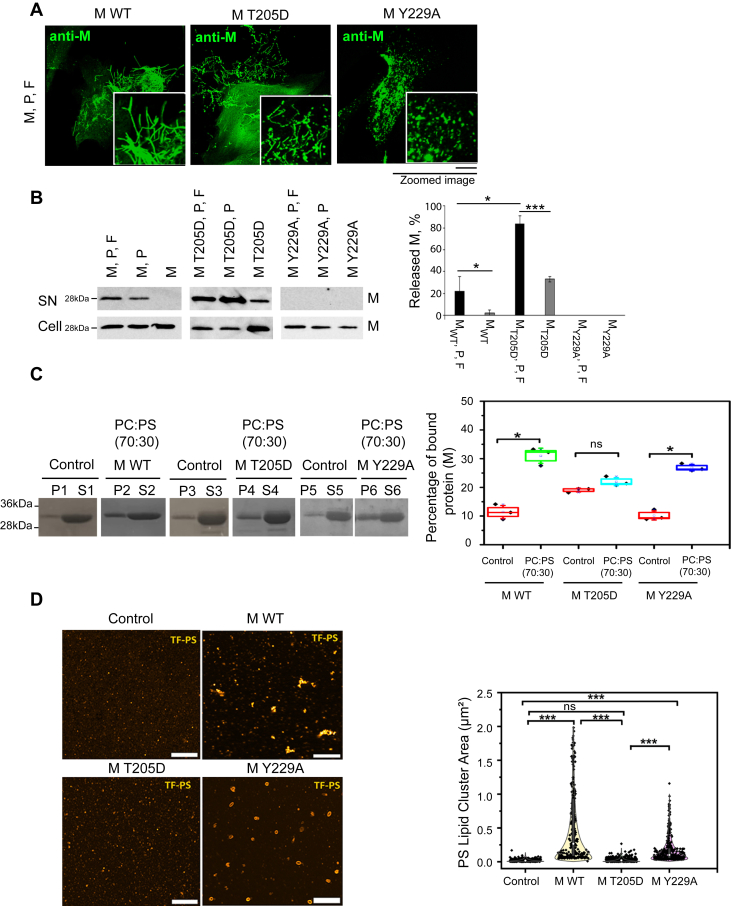
**RSV M binds PS lipids as dimer and M-controlled oligomerization is associated with PS clustering and virus-like filament formation.***A*, epithelial bronchial BEAS-2B cells were cotransfected with plasmids expressing RSV M WT, T205D, or Y229A M mutants, P, and F viral proteins. Cells were fixed, permeabilized at 24 h post transfection, immunostained with an anti-M, and an Alexa Fluor-488 secondary antibody and were imaged by confocal microscopy. Scale bar represents 10 μm. Filaments are zoomed-in. *B*, HEp-2 cells were cotransfected with plasmids expressing RSV M WT, T205D, or Y229A, P and F or a different combination of those. At 48 h post transfection, cell lysates (*bottom*) were generated and VLPs (*top*) were isolated from the supernatant. VLPs and cell lysates were then subjected to Western analysis using an anti-M antibody. The amounts of M protein in VLP fractions were quantified from the Western blots using ImageJ software and are presented as percentage of released M out of total M in both cell lysate and VLP fractions. Data represent the mean percentages ± SD of n = 3 independent experiments. ∗*p* ≤ 0.05, ∗∗*p* ≤ 0.01, ∗∗∗*p* ≤ 0.001 (unpaired two-tailed *t* test). *C*, SDS-PAGE obtained after M-LUV cosedimentation assays and stained with Coomassie blue showing M protein negative control (P1,S1) (without LUVs), M with PC:PS (70:30) (P2,S2), M T205D negative control (P3,S3) (without any lipid), and M T205D with PC:PS (70:30) (P4,S4) LUVs, M Y229A negative control (P5,S5) (without LUVs), and M Y229A with PC:PS (70:30) (P6,S6) LUVs. The plot is showing the percentage of bound RSV M, M T205D, and M Y229A protein to LUVs, on the *right*. Each point is mean ± SD of n = 3 independent experiments. *D*, high resolutions Airyscan images of SLBs containing 70% PC and 30% PS lipids, with a fluorescent PS lipid (*Top* Fluor-PS, 0.2%) without and with M WT, M T205D, or M Y229A proteins (1 μM). The plot for quantifications of PS lipid clusters size without and with M WT, M T205D, or M Y229A proteins is shown (number of clusters N = 350, 201, 280, and 300 for SLBs control without M, with M WT, with M Y229A, and with M T205D, respectively) (each point is a cluster, n = 3 independent experiments). Scale bar represents 5 μm. One-way ANOVA and Mann–Whitney test was used for group comparisons. ns, *p* > 0.05, ∗*p* ≤ 0.05, ∗∗*p* ≤ 0.01, ∗∗∗*p* ≤ 0.001. LUV, large unilamellar vesicle; PC, phosphatidylcholine; PS, phosphatidylserine; RSV, respiratory syncytial virus; SLB, supported lipid bilayer; VLP, virus-like particle.

Next, we investigated whether M oligomerization affects lipid binding and whether M dimer is the major form that interacts with lipids. For that, we used two previously described RSV M mutants, M T205D and M Y229A. M T205D with a phosphomimetic substitution forms dimers that are not stable and higher-order oligomers/aggregates are quickly induced (4). M Y229A forms dimers which are deficient in the formation of higher-order M oligomers and filament formation (19). Purification profiles of the two mutant recombinant M proteins as compared to M WT are shown in Fig. S3. Recombinant RSV with M T205D or M Y229A mutation cannot be recovered as a virus (4, 19). However, RSV virus-like filaments can be generated independently of RSV infection by transfecting cells with plasmids encoding the M, P, and F proteins for virus-like filaments (10, 15) and M and P for VLPs (17) formation. We thus used this assay to compare M WT, M T205D, and M Y229A virus-like filaments formation. Bronchial epithelial BEAS-2B cells were transfected with M WT, M T205D, or M Y229A, P, and F, and the formation of virus-like filaments was assessed using confocal microscopy imaging after staining with a monoclonal anti-M antibody (Fig. 6*A*). Transfection of M WT, P, and F resulted in virus-like filament formation, as reported previously (10, 12). Zoomed-in images are shown focusing on the filaments. Transfection of M T205D, P, and F also formed virus-like filaments, but they seemed more branched and disorganized, as previously reported (4). Transfecting cells with M Y229A, P, and F resulted in short protrusions only, in agreement to what was previously published (19). The lack of virus-like filament formation when using M Y229A reflects the oligomerization defect of this M mutant. Next, we performed a VLP-budding assay (Fig. 6*B*). HEp-2 cells were transfected to express M WT, Y229A, or T205D alone or with P- and F-expressing plasmids. Cell lysates (soluble fraction) and the VLPs released into the cell supernatant were analyzed by Western blotting using anti-M antibodies. Transfecting cells with M, P, and F resulted in the release of VLPs for both WT and T205D M. In the presence of M and P alone, VLPs were also detected, confirming that the two proteins form the minimal platform for RSV VLP budding (17). In contrast to M WT, the expression of M T205D alone resulted in the presence of M in the supernatant (Fig. 6*B*, left panel). Cell expression of M Y229A with or without P and F failed to produce VLPs. VLP-budding efficiency of M WT and M T205D was quantified by western blots and presented as percentage of released M out of total M in both cell lysate and supernatant fractions (Fig. 6*B*, right panel). Our results show that M T205D, when expressed with P and F, is released into the cell supernatant fraction more efficiently than the WT, on average 83.6% *versus* 22.3% VLP release. Moreover, while expression of M WT alone results only in a minor % of M (2.2%) in the supernatant fraction, significantly higher amounts of released M T205D (33.1%) are found, indicating an abnormal release.

We next investigated whether the two M mutants were still able to interact with PS lipids as we identified for M WT (Figure 1, Figure 2, Figure 3, Figure 4, Figure 5). Here, we used the M-LUV cosedimentation assays with PC:PS (70:30) LUVs and measured the presence of M in the P fraction (Fig. 6*C*). In the absence of liposomes (negative control), 10.2, 22.4, and 11.4%, of M WT, M T205D, and M Y229A were found in the P fraction, respectively. When M WT or M Y229A was incubated with PC:PS LUVs, M was found enriched in the P fraction (31.1% and 26.9%), indicating interaction with PS lipids. In contrast, when M T205D was incubated with PC:PS LUVs, there was no significant difference between the negative control and the protein in the presence of PC:PS LUVs, suggesting no interaction (Fig. 6*C*). These results demonstrate that both RSV M WT and M Y229A proteins interact with PC:PS LUVs, suggesting that RSV M can interact with PS lipids as a dimer. M T205D does not interact with PC:PS LUVs, presumably due to its unstable dimer form which oligomerizes/aggregates extensively (4).

To further confirm this result, using AiryScan confocal microscopy, we imaged and analyzed the effect of M T205D and Y229A on PS clustering using PC:PS (70:30) SLBs system with the fluorescent PS lipid probe (Fig. 6*D*). There were no or very few PS clusters in the absence of M protein (negative control): PS cluster size was 0.02 μm^2^. Adding 1 μM M WT induced PS cluster formation (PS cluster size is 0.54 μm^2^), similarly to what is shown in Figure 5. In contrast, there was no significant change in PS clusters size observed after addition of 1 μM M T205D protein (clusters size 0.02 μm^2^), as compared to negative control, confirming no interaction of this M mutant with PC:PS lipidic membranes. Adding 1 μM of M Y229A protein induced PS cluster formation but with a different appearance (clusters size 0.18 μm^2^) as compared to M WT, forming round shape ring-like clusters of PS, suggesting an abnormal protein oligomerization. In conclusion, our results show that the M T205D mutant with excessive oligomerization is not able to bind PC:PS LUVs and to induce clustering of the PS lipids *in vitro* on model membranes as compared to WT RSV M. In contrast, M Y229A mutant, which forms dimers but does not oligomerize properly, binds PC:PS LUVs, inducing PS clustering with different size and appearance as compared to WT M protein.

## Discussion

In this work, we have found that PS lipids mediate the interaction between RSV M protein and host membrane (Fig. 1). It was previously proposed that an extensive contiguous area of positive surface charge on the M monomer drives the interaction with any negatively charged membrane surface (37). Our work here has shown that the interactions between the RSV M protein and the lipid bilayer are indeed electrostatic in nature (Fig. 1, *C* and *D*), but we have also specifically identified the PS as the main drive for M binding to lipid membrane. We have also shown that RSV M is able to cluster PS lipids on model membrane (Fig. 5), most probably a signature of RSV M oligomerization (Fig. 6) on lipid membranes, in a cooperative manner (Fig. 2), as it was also proposed for influenza A virus M1 protein (46).

It was previously reported that several retroviral M proteins could interact with PS with low *K*_d_ at physiological salt concentrations (30). The binding of retroviral matrix proteins to lipids was considered to be purely electrostatic, as the interaction with PS was inhibited at high ionic strength, as we reported in this study for RSV M (Figs. 1, 2 and S2). The reported apparent *K*_d, app_ value for RSV M binding to PS lipids is rather low, but the cooperative n value is superior to 1, indicating a cooperative binding of RSV M upon multimerization on PS lipid.

In comparison to the RSV M protein, several other viruses also have matrix proteins that bind to negative lipids on plasma membranes and induce lipid clustering. Like RSV M, the matrix proteins of the Ebola, Marburg, paramyxovirus Nipah, and measles viruses also interact selectively with PS lipids but, unlike RSV M, PI(4,5)P2 is also required for proper lipid clustering (25, 28, 47). Similarly, HIV matrix protein has also been shown to bind PS but requires PI(4,5)P2 in order to induce cluster formation (32). Our results show that RSV M selectively interacts with PS but not with PE or PG (Fig. 1*B*). Surprisingly, adding low concentration of PI(4, 5)P2 to PC:PS lipids does not increase M binding (Fig. 3*A*). In fact, the RSV M protein interacts similarly to the M1 matrix protein of influenza A virus, which only requires PS for interaction with lipids and for clustering (33, 48).

We have also investigated whether the presence of cholesterol affects M binding to PS lipid membranes (Figs. 3*B* and 5*B*). We have shown that cholesterol has a negative effect on RSV M binding to PS LUVs and reduces PS clustering by M (Figs. 3*B* and 5*B*), possibly indicating a decrease in RSV M multimerization capacity on lipidic membranes. One hypothesis could be that cholesterol provides rigidify (lipid ordering) in the membrane and prevents cooperative multimerization of RSV M on PS membranes, indicating that RSV M might prefer disordered membrane for assembly. However, in RSV-infected cells, M was shown to be associated with lipid nanodomains (40, 49, 50). Moreover, M interacts with Caveolae proteins, Cav-1 and Cav-2 (51), which are the major components of lipid rafts together with cholesterol. However, the sorting of M into lipid rafts was shown to be dependent on the presence of cell surface glycoproteins. In the absence of glycoproteins, M was still found on the plasma membrane but not concentrated in lipid rafts (36). Our results have shown that increasing cholesterol concentration in LUVs prevents M interaction with lipids (Fig. 3*B*). Additionally, the PS clusters size was substantially smaller in the presence of cholesterol (Fig. 5*B*). Although RSV budding, when all the viral proteins assemble together, was suggested by others to occur at lipid rafts (36, 40, 50, 51), our results challenge this claim. The initial M binding may occur elsewhere on the plasma membrane, most probably at PS lipids, then inducing PS clustering upon multimerization, limited or controlled by cholesterol. Our results are comparable to the multimerization of influenza A M1 protein on PS membranes where oligomerization of M1 was favored upon binding to lipid membranes (46). Similarly, HIV Gag was shown to generate his own specific nanoclusters enriched in specific lipids and cholesterol rather than targeting pre-existing plasma membrane lipid rafts (31).

M interaction with FCT was often suggested but never demonstrated directly. Here, we show that the initial binding of the M protein with PS lipids is independent of the FCT protein (Figure 1, Figure 2, Figure 3). This is also in agreement with previously published data (17) and our results in Figure 6*B*, which show M and P being a minimal set for VLP production. M is found in the VLPs in the absence of F. Our results in Figure 4 show that the presence of FCT on LUVs blocks M binding. This suggests that the initial interaction of M with the PS lipids is direct and not *via* the FCT. The helical lattice of M organized as dimers beneath the viral membrane in close proximity to F tails, as seen in the virus, suggests M interaction with FCTs. However, this most probably occurs later and is not required during initial M assembly on the membrane. Importantly, we cannot exclude that the His-GCN4-FCT construct, made to mimic the trimeric FCT, is not properly positioned regarding the distance from the lipid layer since the GCN4 domain results in distancing the FCT from the membrane, and FCT projecting directly from the membrane would mimic more naturally the viral assembly. However, if the GCN4-FCT accurately mimics the trimeric FCT, the results strongly suggest that M must initially bind membranes independently of FCT.

To our knowledge, the question of whether M binds plasma membranes as dimers or as oligomers was not addressed previously. The basic RSV M unit is a dimer (19) and M in the cytoplasm was shown to be dimeric (20). This makes sense, since M is also found in inclusion bodies which are membrane-free viral structures (52, 53). Our results based on RSV M dimerization/oligomerization mutant proteins *in vitro* suggest that M binds PS lipids as dimers (Fig. 6). We used previously published M oligomerization mutants, M T205D and M Y229A, in order to analyze the possible link between M oligomerization and lipid binding and clustering. M T205D, which oligomerizes/aggregates extensively, formed virus-like filaments but they were more branched and disorganized than M WT. Virus-like filament formation indicates that M T205D is able to bind to plasma membrane but possibly due to wrong protein–lipid interactions. In contrast, M Y229A, which is mostly dimeric, did not for filaments (Fig. 6*A*). Using VLP assay, we showed that expression of M T205D results in significantly higher amount of released M from cells, also when no other viral protein was present. In contrast, expression of M Y229A prevented VLP production (Fig. 6*B*). M T205D forms dimers which quickly induce higher-order oligomers and aggregates (4). We cannot exclude that also the dimer formed by this mutant is differently folded, and this is the cause of extensive oligomerization/aggregation. Previously, M T205D-induced virus-like filaments were shown to be shorter and not regular than WT M forming filaments. Staining with anti M antibody clearly showed that M T205D formed bulky looking aggregates, in contrast to WT M which appeared regular and equally distributed along the filaments (4). This could possibly explain the presence of M T205D in the supernatant. RSV is known to be mostly cell-associated and the VLP release to be relatively inefficient. Our VLP assay using WT M shows that only about 20% M is found in the supernatant, presumably in VLPs. In contrast, when using M T205D, with other viral proteins or alone, relatively high percentage of M was found in the supernatant. Our hypothesis is that M T205D oligomers/aggregates in the filaments cause extensive tension on the lipids and eventually breaking off the filaments, which results in increased percentage of M in the supernatant.

M WT and M Y229A bound PC:PS LUVs in a similar way, contrary to M T205D mutant which was similar to negative control without LUVs (Fig. 6*C*). This was confirmed using SLBs; no clustering of PS lipids with M T205D was detected. In this assay, however, different clusters of PS were seen between M WT and M Y229A, the mutant-forming smaller clusters (Fig. 6*D*). We therefore conclude, based on our results and published work, that M binds to plasma membrane lipids as dimers and only forms higher-order oligomers during viral filament formation and VLPs budding. Proper M oligomerization is associated with PS clustering. Similar results were shown also for other viral matrix proteins, for example EBOV and MARV VP40 (24).

In conclusion, the results presented above demonstrate the selective binding of the RSV M protein to PS lipids and its ability to induce lipid clustering. This is similar to other enveloped viruses, highlighting the importance of matrix–PS lipid interaction for enveloped viruses which bud from the plasma membrane.

## Experimental procedures

### Cell culture

HEp-2 cells were maintained in Dulbecco’s modified Eagle’s medium (eurobio) supplemented with 10% fetal calf serum (eurobio), 1% L-glutamine, and 1 % penicillin streptomycin. The transformed human bronchial epithelial cell line (BEAS-2B) (ATCC CRL-9609) was maintained in RPMI 1640 medium (eurobio) supplemented with 10% fetal calf serum, 1% L-glutamine, and 1% penicillin-streptomycin. The cells were grown at 37 °C in 5% CO_2_.

### Plasmids

pCDF plasmids encoding RSV M or GCN4-FCT proteins were used for the expression and purification of recombinant M and GCN4-FCT proteins, and pcDNA3.1 codon-optimized plasmids encoding the RSV M, P, and F proteins (gift from M. Moore, Emory University) (54) were used for the expression of viral proteins in cells. M Y229A and MT205D substitutions were generated using the Quick change directed mutagenesis kit (New England Biolabs), as recommended by the manufacturer.

### Bacteria expression and purification of recombinant proteins

For M expression (WT, T205D, and Y229A mutant), *Escherichia coli* Rosetta 2 bacteria transformed with the pCDF-M plasmid were grown from fresh starter cultures in LB broth for 5 h at 32 °C, followed by induction with 0.4 mM IPTG for 4 h at 25 °C. Cells were lysed by sonication (3 times for 20 s each time) and lysozyme (1 mg/ml; Sigma) in 50 mM NaH_2_PO_4_-Na_2_HPO_4_, 300 mM NaCl, pH 7.4, plus protease inhibitors (Roche), RNase (12 g/ml, Sigma), and 0.25% CHAPS {3-[(3-cholamidopropyl)-dimethylammonio]-1-propanesulfonate}. Lysates were clarified by centrifugation (23,425*g*, 30 min, 4 °C), and the soluble His_6_-M protein was purified on a Nickel sepharose column (HiTrap 5 ml IMAC HP; GE Healthcare). The bound protein was washed extensively with loading buffer plus 25 mM imidazole and eluted with a 25 to 250 mM imidazole gradient. M was concentrated to 2 ml using Vivaspin20 columns (Sartorius Stedim Biotech) and purified on a HiLoad 10/600 Superdex S200 column (GE Healthcare) in 50 mM NaH_2_PO_4_-Na_2_HPO_4_, 300 mM NaCl, pH 7.4. The M peak was concentrated to 3 mg/ml using Vivaspin4 columns. The His tag was digested during 14 h at 4 °C with His-tagged 3C proteases (Thermo Fisher Scientific) according to the manufacturer’s recommendations, incubated for 40 min at 4 °C with nickel phosphate–loaded Sepharose beads (GE Healthcare) to separate the His tag, and concentrated using 10,000 MWCO Vivaspin20 columns. The quality of protein samples was assessed by SDS-PAGE. Protein concentration was determined by measuring absorbance at 280 nm.

For expression of recombinant His-GCN4-FCT, *E. coli* BL21 bacteria were transformed with pCDF-FCT plasmid, grown in LB broth for 5 h at 32 °C, and then induced with 0.5 mM final IPTG for 4 h at 25 °C. Bacteria were resuspended in lysis buffer (50 mM NaH_2_PO_4_-Na_2_HPO_4_, 300 mM NaCl, pH 7.2) supplemented with 1 mg/ml lysozyme (Sigma) and 10 mg/ml protease inhibitors (Roche) and then lysed by sonication (3 times for 20 s each time). The lysates were clarified by centrifugation (23,425*g*, 30 min, 4 °C) and the soluble proteins with His_6_ tag were purified on 1 ml of nickel phosphate–loaded sepharose beads (GE Healthcare). Bound proteins were washed in lysis buffer with 25 mM imidazole then with 400 mM Imidazole, eluted with 800 mM imidazole, and concentrated to 2 ml using MWCO Vivaspin4 5000 columns (Sartorius Stedim Biotech). His-GCN4-FCT was further purified on a HiLoad 10/600 Superdex S200 column (GE Healthcare) in 50 mM NaH_2_PO_4_-Na_2_HPO_4_, 300 mM NaCl, pH 7.2. Proteins were concentrated to 2 ml final using MWCO Vivaspin4 5000 columns. Purification profiles of M WT, M T205D, M Y229A, and His-GCN4-FCT protein are shown in Fig. S3.

### LUVs preparations

Avanti Polar Lipids supplied all of the lipids used in this study. Using chloroform, lipid mix with the appropriate ratio compositions was created, and lipid mixture solutions were dried to make lipid films using a vacuum pump coupled rotavapor. Each experiment compensated for the addition of anionic lipids/cholesterol with an equal mol% decrease in Egg-PC. A phospholipid assay kit (Sigma Aldrich) was used to determine the concentration of the lipid stock solution. After incubation at 25 °C, lipid films were hydrated in Tris-buffer (150 mM NaCl, 10 mM Tris–HCl, pH 7.4) using freeze-thaw cycles and extruded through a 100/200-nm Whatman polycarbonate filter (GE Healthcare). The size of the LUVs was confirmed using dynamic light scattering on a Malvern Panalytical Zetasizer Nano.

### *In vitro* cosedimentation assays with LUVs

A required amount of desired protein was incubated with LUVs of 200 nm (1 mg/ml) in a final volume of 100 μl at 25 °C for 30 min. Samples were then centrifuged at 220,000*g* in a Beckman TLA 100 rotor at 4 °C for 30 min. Each sample was then divided into supernatant (S = 90 μl), containing unbound protein, and pellet (*p* = 10 μl), containing LUV-bound protein. P was diluted in 80 μl of Tris–NaCl buffer (150 mM NaCl, 10 mM Tris–HCl, pH 7.4) buffer to maintain the equivalence between the S and P volumes. Twenty microliters of S and P were analyzed by SDS-PAGE and protein were detected by staining with Coomassie Blue. The full SDS-PAGE Coomassie gels are shown in Figs. S4 and S5. For Ni-incorporated LUVs preparation, 1,2-dioleoyl-sn-glycero-3-[(N-(5-amino-1-carboxypentyl) iminodiacetic acid) succinyl] (nickel salt) lipids were used. The protein intensities (intensity of supernatant: I_s_, intensity of pellet: I_p_) were quantified using the Image J software (55). The percentage of LUV-bound protein was calculated as:

(Equation 1) $$\text{Percentage}\;\text{of}\;\text{protein}\;\text{bound}\;\text{to}\;\text{LUVs}=100*I_{P}/\left(I_{P}+I_{S}\right)$$

### *In vitro* lipid flotations assay with LUVs

LUVs of desired lipids compositions were prepared as described above. LUVs (200 nm) (1 mg/ml) and protein were incubated for 30 min in Tris-buffer (150 mM NaCl, 10 mM Tris–HCl, pH 7.4) at 25 °C. LUVs and protein (50 μl) were then mixed with Tris-buffer supplemented with 60% sucrose (75 μl) and then overlaid with an intermediate sucrose solution made of Tris-buffer with 25% sucrose (75 μl) and a layer (25 μl) of Tris-buffer. After centrifugation at 100,000 rpm in a fixed-angle TLA100 rotor for 1 h, three fractions (bottom, medium, and top) of 125, 50, and 50 μl were collected. Top and bottom layers were analyzed by SDS-PAGE and staining with Coomassie Blue. The protein intensities (intensity of top layer: I_T_, intensity of bottom layer: I_B_) were quantified using the Image J software.

(Equation 2) $$\text{Percentage}\;\text{of}\;\text{protein}\;\text{bound}\;\text{to}\;\text{LUVs}=100*I_{T}/\left(I_{T}+I_{B}\right)$$

### Binding affinity determinations

We have estimated an apparent binding constant “*K*_d, app_” to characterize the binding of protein to lipid, as described previously (56). K is the proportionality constant between the molar concentration of the protein in the bulk aqueous phase, [RSV M]_F_, and the fraction of proteins bound to the lipids, [RSV M]_B_.

The fraction of protein bound is given by.

(Equation 3) $${\left[\text{RSV}\;M\right]}_{B}={\left[\text{RSV}\;M\right]}_{\max}\;\frac{K\left[\text{PS}\right]\text{acc}}{1+K\left[\text{PS}\right]\text{acc}}$$

[PS]acc is the concentration of accessible PS. Here, it is assumed, roughly, that all PS on the outer leaflet is accessible. The apparent dissociation constant, also known as the apparent association constant *K*_d, app_, is the reciprocal of the constant K, which is inferred from Equation 3. Existence of cooperativity in the RSV M binding to the different mol percent PS containing LUVs are derived using Hill equations as described previously (57).

(Equation 4) $${\left[\text{RSV}\;M\right]}_{B}=\frac{{\left[\text{PS}\right]\text{acc}}^{n}}{\text{Kd}+{\left[\text{PS}\right]\text{acc}}^{n}}$$

Where n = Hill coefficient.

### SLB preparation

Vesicle fusion method for SLBs preparation was used, as previously described (58). The cover slips were cleaned with piranha solution (sulfuric acid (H2SO4): hydrogen peroxide (H_2_O_2_) 3:1). LUVs of 100 nm with desired lipid compositions and with fluorescent lipid in Tris (150 mM NaCl, 10 mM Tris–HCl, pH 7.4) buffer were prepared according to method describe above. 0.5 mg/ml of LUVs in the cleaned glass cover slips were deposited and kept it in 55 °C for 30 min for SLBs preparation. The coverslips were washed with Tris-buffer (150 mM NaCl, 10 mM Tris–HCl, pH 7.4) to clean unfused vesicles. Desired protein concentrations were directly added to the surface of SLBs and incubated for 30 min for each condition. Confocal and Airy scan fluorescence images were generated using a LSM980-laser-scanning microscope (Zeiss) equipped with a 63×, 1.4 NA oil objective. All the images were processed with ImageJ/Fiji software. The PS-lipid clusters area for different conditions were measured using image J segmentation tool (ImageJ/Fiji).

### Virus-like filament/particle formation

Overnight cultures of BEAS-2B cells seeded at four 10^5^ cells/well in 6-well plates (on a 16-mm microcover glass for immunostaining) were transfected with pcDNA3.1 codon-optimized plasmids (0.4 μg each) carrying the RSV A2 WT, T205D, or Y229A M along with pcDNA3.1 codon-optimized plasmids carrying RSV A2 P and F using Lipofectamine 2000 (Invitrogen) according to the manufacturer’s recommendations. Cells were fixed 24 h post transfection, immunostained, and imaged as described below. For VLP formation, overnight cultures of HEp-2 cells seeded at four 10^5^ cells/well in 6-well plates were transfected as described above. Released VLPs were harvested from the supernatant; the supernatant was clarified of cell debris by centrifugation (1,300*g*, 10 min, 4° C) and pelleted through a 20% sucrose cushion (13,500*g*, 90 min, 4 °C). Cells were lysed in radio immune precipitation assay buffer (0.5 M Tris–HCl, pH 7.4, 1.5 M NaCl, 2.5% deoxycholic acid, 10% NP-40, 10 mM EDTA). Cellular lysates and VLP pellets were dissolved in Laemmli buffer (250 mM Tris–HCl (pH 6.8), 8% SDS, 40% glycerol, 8% beta-mercaptoethanol, and 0.02% bromophenol blue) and subjected to Western analysis. The full Western Blots are shown in Fig. S6. The amounts of M protein in VLP fractions were quantified using the ImageJ software and are presented as percentage of released M out of total M in both cell lysate and supernatant fractions.

### SDS-PAGE and western analysis

Protein samples were separated by electrophoresis on 12% polyacrylamide gels in Tris-glycine buffer. All samples were boiled for 3 min prior to electrophoresis. Proteins were then transferred to a nitrocellulose membrane (Roche Diagnostics). The blots were blocked with 5% nonfat milk in Tris-buffered saline (pH 7.4), followed by incubation with rabbit anti-M antiserum (1:1000) or mouse anti-His antibody (1:1000) (Invitrogen) and horseradish peroxidase–conjugated donkey anti-rabbit (1:10,000) or anti-mouse (1:10,000) antibodies (P.A.R.I.S.). Western blots were developed using freshly prepared chemiluminescent substrate (100 mM Tris–HCl, pH 8.8, 1.25 mM luminol, 0.2 mM p-coumaric acid, 0.05% H_2_O_2_) and exposed using BIO-RAD ChemiDoc Touch Imaging System.

### Generation of M antiserum

Polyclonal anti M serum was prepared by immunizing a rabbit three times at 2-weeks intervals using purified His-fusion proteins (100 mg) for each immunization. The first and second immunizations were administered subcutaneously in 1 ml Freund’s complete and Freund’s incomplete adjuvant (Difco), respectively. The third immunization was done intramuscularly in Freund’s incomplete adjuvant. Animals were bled 10 days after the third immunization.

### Immunostaining and imaging

Cells were fixed with 4% paraformaldehyde in PBS for 10 min, blocked with 3% bovine serum albumin in 0.2% Triton X-100–PBS for 10 min, and immunostained with monoclonal anti-M (1:200; a gift from Mariethe Ehnlund, Karolinska Institute), followed by species-specific secondary antibodies conjugated to Alexa Fluor 488 (1: 1000; Invitrogen). Images were obtained using the White Light laser SP8 (Leica Microsystems) or the Zeiss LSM700 confocal microscope at a nominal magnification of 63× oil. Images were acquired using the Leica Application Suite X (LAS X) software (https://www.leica-microsystems.com/products/microscope-software).

## Data availability

All data are contained within the article or supporting information.

## Supporting information

This article contains supporting information.

## Conflicts of interest

The authors declare that they have no conflict of interest with the contents of this article.

## References

[1] Coultas J.A., Smyth R., Openshaw P.J. (2019). Respiratory syncytial virus (RSV): a scourge from infancy to old age. Thorax.

[2] Afonso C.L., Amarasinghe G.K., Banyai K., Bao Y., Basler C.F., Bavari S. (2016). Taxonomy of the order mononegavirales: update 2016. Arch. Virol..

[3] Ke Z., Dillard R.S., Chirkova T., Leon F., Stobart C.C., Hampton C.M. (2018). The morphology and assembly of respiratory syncytial virus revealed by cryo-electron tomography. Viruses.

[4] Bajorek M., Caly L., Tran K.C., Maertens G.N., Tripp R.A., Bacharach E. (2014). The Thr205 phosphorylation site within respiratory syncytial virus matrix (M) protein modulates M oligomerization and virus production. J. Virol..

[5] Vanover D., Smith D.V., Blanchard E.L., Alonas E., Kirschman J.L., Lifland A.W. (2017). RSV glycoprotein and genomic RNA dynamics reveal filament assembly prior to the plasma membrane. Nat. Commun..

[6] Vijayakrishnan S., Burns A.M., Blanchard E.L., Spink M.C., Gilchrist J., Howe A. (2022). Ultrastructural characterization of a viral RNA and G-protein containing, membranous organelle formed in respiratory syncytial virus infected cells. bioRxiv.

[7] Roberts S.R., Compans R.W., Wertz G.W. (1995). Respiratory syncytial virus matures at the apical surfaces of polarized epithelial cells. J. Virol..

[8] Liljeroos L., Krzyzaniak M.A., Helenius A., Butcher S.J. (2013). Architecture of respiratory syncytial virus revealed by electron cryotomography. Proc. Natl. Acad. Sci. U. S. A..

[9] Meshram C.D., Baviskar P.S., Ognibene C.M., Oomens A.G. (2016). The respiratory syncytial virus phosphoprotein, matrix protein, and fusion protein Carboxy-terminal domain drive efficient filamentous virus-like particle formation. J. Virol..

[10] Bajorek M., Galloux M., Richard C.A., Szekely O., Rosenzweig R., Sizun C. (2021). Tetramerization of phosphoprotein is essential for respiratory syncytial virus budding while its N terminal region mediates direct interactions with the matrix protein. J. Virol..

[11] Harrison M.S., Sakaguchi T., Schmitt A.P. (2010). Paramyxovirus assembly and budding: building particles that transmit infections. Int. J. Biochem. Cell Biol..

[12] Mitra R., Baviskar P., Duncan-Decocq R.R., Patel D., Oomens A.G. (2012). The human respiratory syncytial virus matrix protein is required for maturation of viral filaments. J. Virol..

[13] Kiss G., Holl J.M., Williams G.M., Alonas E., Vanover D., Lifland A.W. (2014). Structural analysis of respiratory syncytial virus reveals the position of M2-1 between the matrix protein and the ribonucleoprotein complex. J. Virol..

[14] Conley M.J., Short J.M., Burns A.M., Streetley J., Hutchings J., Bakker S.E. (2022). Helical ordering of envelope-associated proteins and glycoproteins in respiratory syncytial virus. EMBO J..

[15] Baviskar P.S., Hotard A.L., Moore M.L., Oomens A.G. (2013). The respiratory syncytial virus fusion protein targets to the perimeter of inclusion bodies and facilitates filament formation by a cytoplasmic tail-dependent mechanism. J. Virol..

[16] Shaikh F.Y., Cox R.G., Lifland A.W., Hotard A.L., Williams J.V., Moore M.L. (2012). A critical phenylalanine residue in the respiratory syncytial virus fusion protein cytoplasmic tail mediates assembly of internal viral proteins into viral filaments and particles. MBio.

[17] Ha B., Yang J.E., Chen X., Jadhao S.J., Wright E.R., Anderson L.J. (2020). Two RSV platforms for G, F, or G+F proteins VLPs. Viruses.

[18] Ghildyal R., Ho A., Jans D.A. (2006). Central role of the respiratory syncytial virus matrix protein in infection. FEMS Microbiol. Rev..

[19] Forster A., Maertens G.N., Farrell P.J., Bajorek M. (2015). Dimerization of matrix protein is required for budding of respiratory syncytial virus. J. Virol..

[20] Trevisan M., Di Antonio V., Radeghieri A., Palu G., Ghildyal R., Alvisi G. (2018). Molecular requirements for self-interaction of the respiratory syncytial virus matrix protein in living mammalian cells. Viruses.

[21] Chen B.J., Lamb R.A. (2008). Mechanisms for enveloped virus budding: can some viruses do without an ESCRT?. Virology.

[22] van Meer G., Voelker D.R., Feigenson G.W. (2008). Membrane lipids: where they are and how they behave. Nat. Rev. Mol. Cell Biol..

[23] Fujimoto T., Parmryd I. (2016). Interleaflet coupling, pinning, and leaflet asymmetry-major players in plasma membrane nanodomain formation. Front. Cell Dev. Biol..

[24] Motsa B.B., Stahelin R.V. (2021). Lipid-protein interactions in virus assembly and budding from the host cell plasma membrane. Biochem. Soc. Trans..

[25] Adu-Gyamfi E., Johnson K.A., Fraser M.E., Scott J.L., Soni S.P., Jones K.R. (2015). Host cell plasma membrane phosphatidylserine regulates the assembly and budding of Ebola virus. J. Virol..

[26] Johnson K.A., Taghon G.J., Scott J.L., Stahelin R.V. (2016). The Ebola Virus matrix protein, VP40, requires phosphatidylinositol 4,5-bisphosphate (PI(4,5)P2) for extensive oligomerization at the plasma membrane and viral egress. Sci. Rep..

[27] Amiar S., Husby M.L., Wijesinghe K.J., Angel S., Bhattarai N., Gerstman B.S. (2021). Lipid-specific oligomerization of the Marburg virus matrix protein VP40 is regulated by two distinct interfaces for virion assembly. J. Biol. Chem..

[28] Norris M.J., Husby M.L., Kiosses W.B., Yin J., Saxena R., Rennick L.J. (2022). Measles and Nipah virus assembly: specific lipid binding drives matrix polymerization. Sci. Adv..

[29] Mercredi P.Y., Bucca N., Loeliger B., Gaines C.R., Mehta M., Bhargava P. (2016). Structural and molecular determinants of membrane binding by the HIV-1 matrix protein. J. Mol. Biol..

[30] Hamard-Peron E., Muriaux D. (2011). Retroviral matrix and lipids, the intimate interaction. Retrovirology.

[31] Yandrapalli N., Lubart Q., Tanwar H.S., Picart C., Mak J., Muriaux D. (2016). Self assembly of HIV-1 Gag protein on lipid membranes generates PI(4,5)P2/Cholesterol nanoclusters. Sci. Rep..

[32] Favard C., Chojnacki J., Merida P., Yandrapalli N., Mak J., Eggeling C. (2019). HIV-1 Gag specifically restricts PI(4,5)P2 and cholesterol mobility in living cells creating a nanodomain platform for virus assembly. Sci. Adv..

[33] Kerviel A., Dash S., Moncorge O., Panthu B., Prchal J., Decimo D. (2016). Involvement of an arginine triplet in M1 matrix protein interaction with membranes and in M1 recruitment into virus-like particles of the influenza A(H1N1)pdm09 virus. PLoS One.

[34] Banerjee S., Aponte-Diaz D., Yeager C., Sharma S.D., Ning G., Oh H.S. (2018). Hijacking of multiple phospholipid biosynthetic pathways and induction of membrane biogenesis by a picornaviral 3CD protein. PLoS Pathog..

[35] Kolli S., Meng X., Wu X., Shengjuler D., Cameron C.E., Xiang Y. (2015). Structure-function analysis of vaccinia virus H7 protein reveals a novel phosphoinositide binding fold essential for poxvirus replication. J. Virol..

[36] Henderson G., Murray J., Yeo R.P. (2002). Sorting of the respiratory syncytial virus matrix protein into detergent-resistant structures is dependent on cell-surface expression of the glycoproteins. Virology.

[37] Money V.A., McPhee H.K., Mosely J.A., Sanderson J.M., Yeo R.P. (2009). Surface features of a Mononegavirales matrix protein indicate sites of membrane interaction. Proc. Natl. Acad. Sci. U. S. A..

[38] McPhee H.K., Carlisle J.L., Beeby A., Money V.A., Watson S.M., Yeo R.P. (2011). Influence of lipids on the interfacial disposition of respiratory syncytical virus matrix protein. Langmuir.

[39] Cho W., Stahelin R.V. (2005). Membrane-protein interactions in cell signaling and membrane trafficking. Annu. Rev. Biophys. Biomol. Struct..

[40] McCurdy L.H., Graham B.S. (2003). Role of plasma membrane lipid microdomains in respiratory syncytial virus filament formation. J. Virol..

[41] Brown G., Aitken J., Rixon H.W., Sugrue R.J. (2002). Caveolin-1 is incorporated into mature respiratory syncytial virus particles during virus assembly on the surface of virus-infected cells. J. Gen. Virol..

[42] Oomens A.G., Bevis K.P., Wertz G.W. (2006). The cytoplasmic tail of the human respiratory syncytial virus F protein plays critical roles in cellular localization of the F protein and infectious progeny production. J. Virol..

[43] McLellan J.S., Chen M., Leung S., Graepel K.W., Du X., Yang Y. (2013). Structure of RSV fusion glycoprotein trimer bound to a prefusion-specific neutralizing antibody. Science.

[44] Harbury P.B., Kim P.S., Alber T. (1994). Crystal structure of an isoleucine-zipper trimer. Nature.

[45] Sliepen K., van Montfort T., Melchers M., Isik G., Sanders R.W. (2015). Immunosilencing a highly immunogenic protein trimerization domain. J. Biol. Chem..

[46] Hilsch M., Goldenbogen B., Sieben C., Hofer C.T., Rabe J.P., Klipp E. (2014). Influenza A matrix protein M1 multimerizes upon binding to lipid membranes. Biophys. J..

[47] Chan R.B., Tanner L., Wenk M.R. (2010). Implications for lipids during replication of enveloped viruses. Chem. Phys. Lipids.

[48] Bobone S., Hilsch M., Storm J., Dunsing V., Herrmann A., Chiantia S. (2017). Phosphatidylserine lateral organization influences the interaction of influenza virus matrix protein 1 with lipid membranes. J. Virol..

[49] Marty A., Meanger J., Mills J., Shields B., Ghildyal R. (2004). Association of matrix protein of respiratory syncytial virus with the host cell membrane of infected cells. Arch. Virol..

[50] Brown G., Rixon H.W., Sugrue R.J. (2002). Respiratory syncytial virus assembly occurs in GM1-rich regions of the host-cell membrane and alters the cellular distribution of tyrosine phosphorylated caveolin-1. J. Gen. Virol..

[51] Kipper S., Hamad S., Caly L., Avrahami D., Bacharach E., Jans D.A. (2015). New host factors important for respiratory syncytial virus (RSV) replication revealed by a novel microfluidics screen for interactors of matrix (M) protein. Mol. Cell. Proteomics.

[52] Galloux M., Risso-Ballester J., Richard C.A., Fix J., Rameix-Welti M.A., Eleouet J.F. (2020). Minimal Elements required for the formation of respiratory syncytial virus cytoplasmic inclusion bodies in Vivo and in vitro. MBio.

[53] Rincheval V., Lelek M., Gault E., Bouillier C., Sitterlin D., Blouquit-Laye S. (2017). Functional organization of cytoplasmic inclusion bodies in cells infected by respiratory syncytial virus. Nat. Commun..

[54] Hotard A.L., Shaikh F.Y., Lee S., Yan D., Teng M.N., Plemper R.K. (2012). A stabilized respiratory syncytial virus reverse genetics system amenable to recombination-mediated mutagenesis. Virology.

[55] Schindelin J., Arganda-Carreras I., Frise E., Kaynig V., Longair M., Pietzsch T. (2012). Fiji: an open-source platform for biological-image analysis. Nat. Methods.

[56] Blin G., Margeat E., Carvalho K., Royer C.A., Roy C., Picart C. (2008). Quantitative analysis of the binding of ezrin to large unilamellar vesicles containing phosphatidylinositol 4,5 bisphosphate. Biophys. J..

[57] Goutelle S., Maurin M., Rougier F., Barbaut X., Bourguignon L., Ducher M. (2008). The Hill equation: a review of its capabilities in pharmacological modelling. Fundam. Clin. Pharmacol..

[58] Seeger H.M., Marino G., Alessandrini A., Facci P. (2009). Effect of physical parameters on the main phase transition of supported lipid bilayers. Biophys. J..

