# Supplementary material for: Selective targeting and clustering of phosphatidylserine lipids by RSV M protein is critical for virus particle production
Source: J Biol Chem. 2023 Oct 5;299(11):105323. doi: 10.1016/j.jbc.2023.105323 (PMC10641529; doi:10.1016/j.jbc.2023.105323)
Supplement: Supporting Figures S1–S6 [file mmc1.pdf]

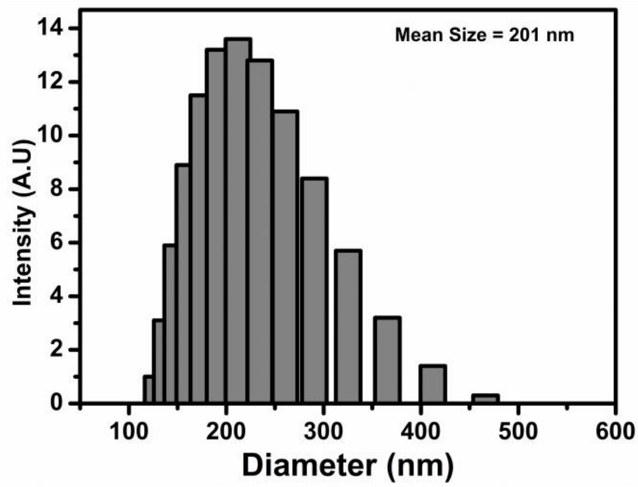

Supplemental Figure 1. Characterization of Egg-PC:Brain-PS (70:30) large unilamellar vesicles (LUVs): Dynamic light scattering (DLS) data plot demonstrates the distribution of LUVs with an average diameter 201 nm and polydispersity index 0.18.

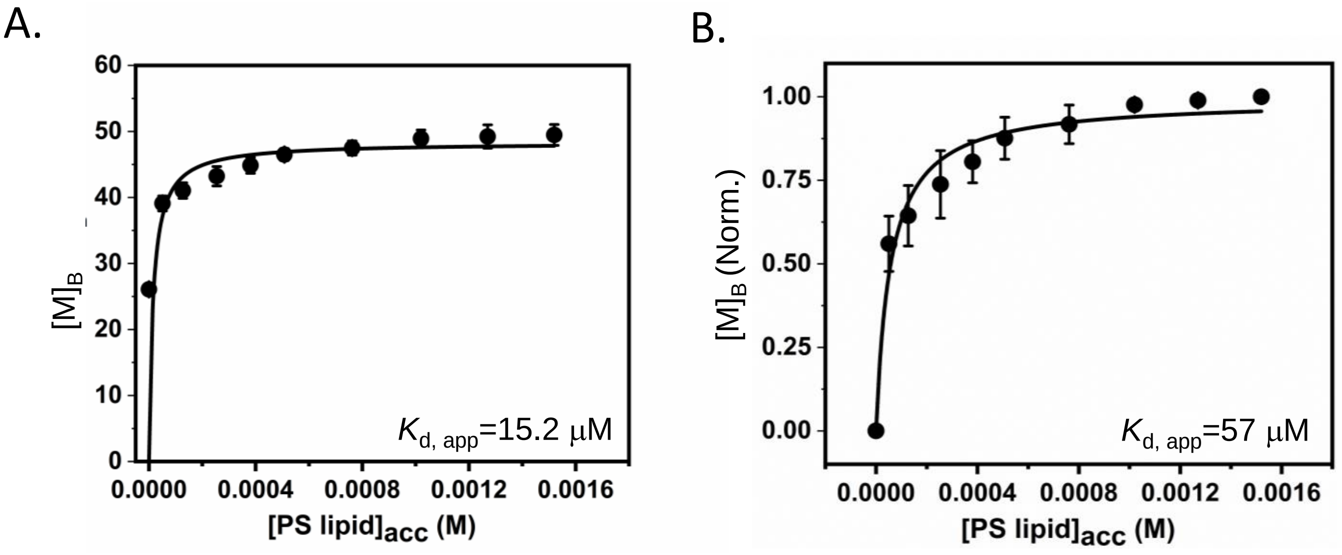

Supplemental Figure 2. A) The plot shows the percentage of M bound ($[M]_B$) (y -axis) with accessible PS lipid concentrations in mol ($[\text{PS lipid}]_{acc}$ (x-axis). B)The plot shows the percentage of M bound ($[M]_B$) normalized (y -axis) with accessible PS lipid concentrations in mol ($[\text{PS lipid}]_{acc}$ (x-axis). Both curves represent the least squares fits of Eq. 3 to the data, which yield the value for the molar partition coefficient K from which the apparent binding constant $K_{d,\ app}$ (Kd= 1/K) can be deduced. Each point is a mean of n=3 independent experiments with standard deviation (SD).

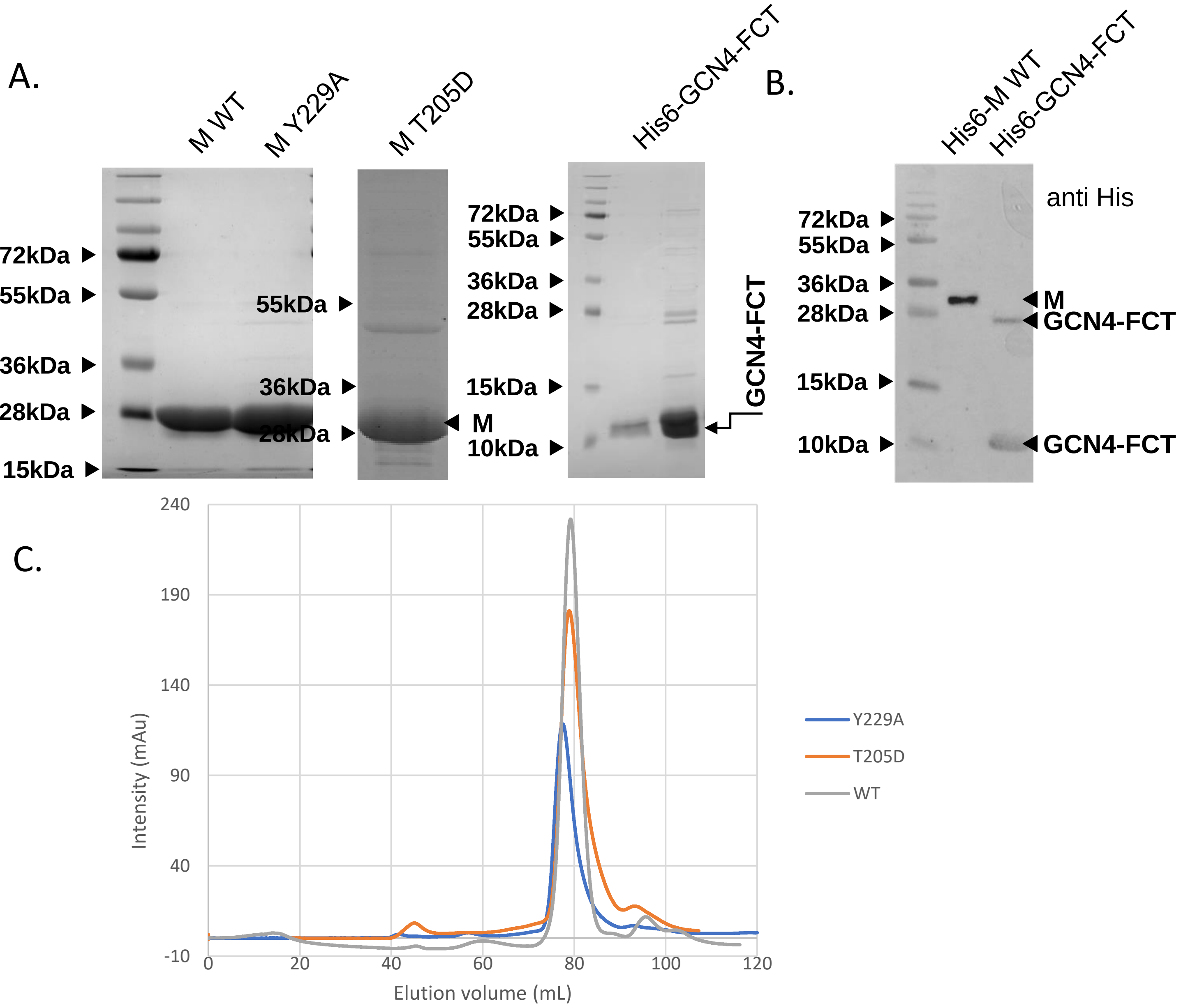

Supplemental Figure 3. Purification profiles for M and FCT proteins purified on Nickel sepharose and HiLoad 10/600 Superdex S200 column as described in Experimental procedures.
A) SDS-PAGE Coomassie blue staining of the final M and GCN4-FCT proteins used. B) SDS-PAGE and Western blot with anti His antibody on His-M WT and His-GCN4-FCT proteins after Nickel purification. C) S200 column elution profile of M WT and mutant proteins.

1B

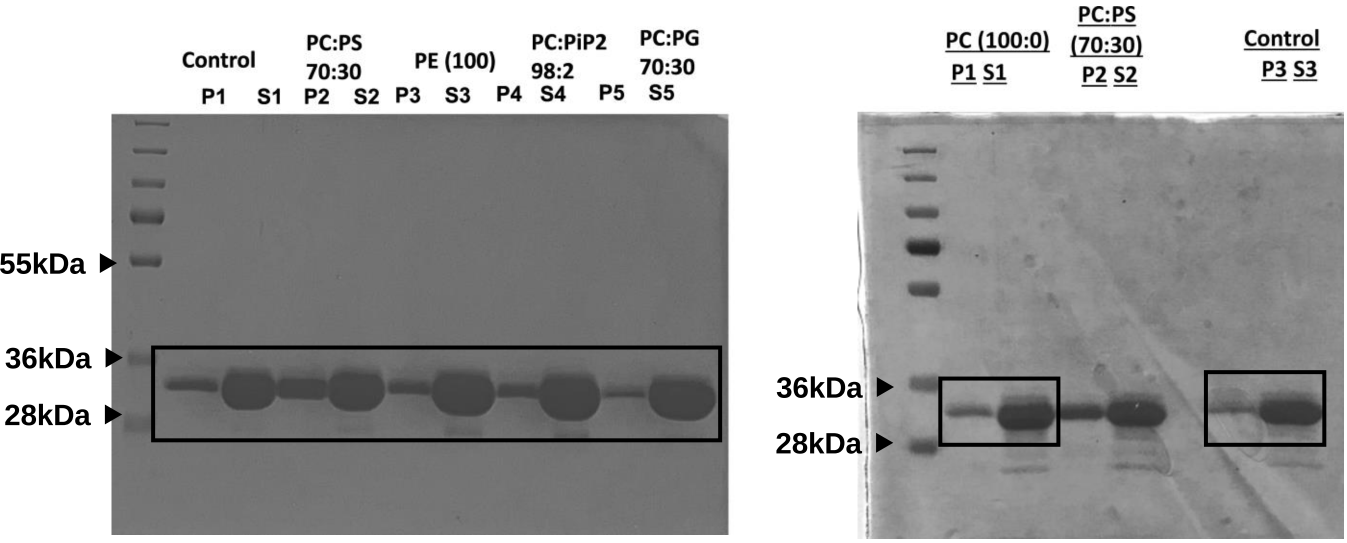

Control
PC:PS 70:30
PE (100)
PC:PiP2 98:2
PC:PG 70:30
P1 S1 P2 S2 P3 S3 P4 S4 P5 S5
55kDa
36kDa
28kDa
PC (100:0)
P1 S1
PC:PS (70:30)
P2 S2
Control
P3 S3
36kDa
28kDa

1C

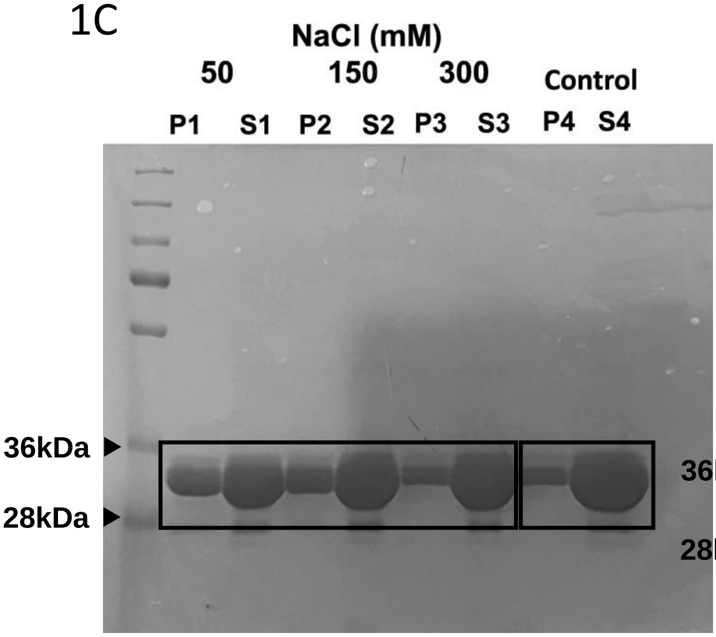

NaCl (mM)
50
150
300
Control
P1 S1 P2 S2 P3 S3 P4 S4
36kDa
28kDa

1D

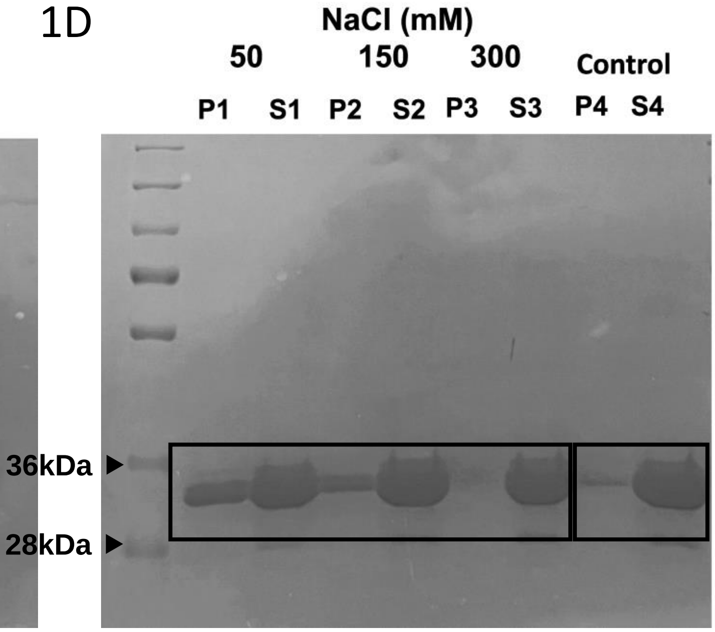

NaCl (mM)
50
150
300
Control
P1 S1 P2 S2 P3 S3 P4 S4
36kDa
28kDa

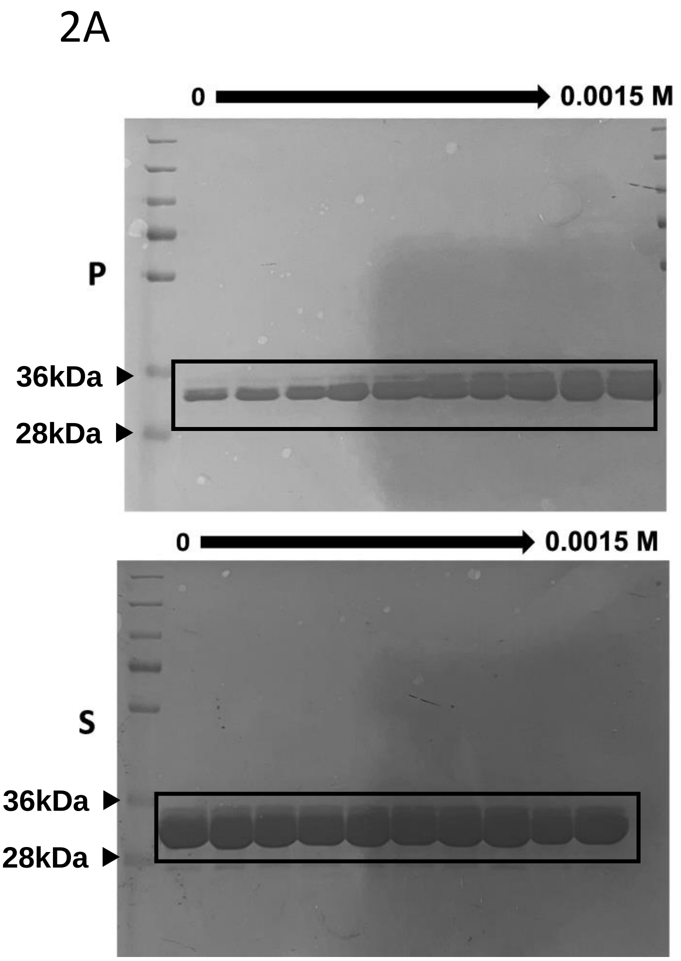

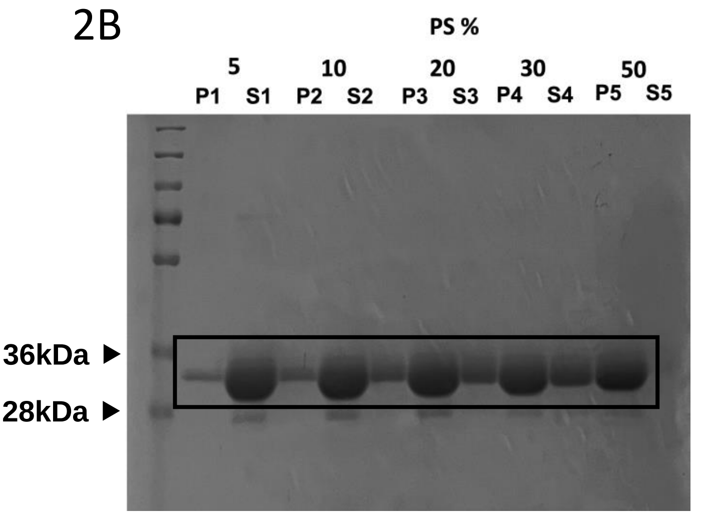

Supplemental Figure 4. Full SDS-PAGE Coomassie gels for 1B, 1C, 1D, 2A, and 2B.
The spliced image panels used in the main figures are marked each on its original gel source.

3A

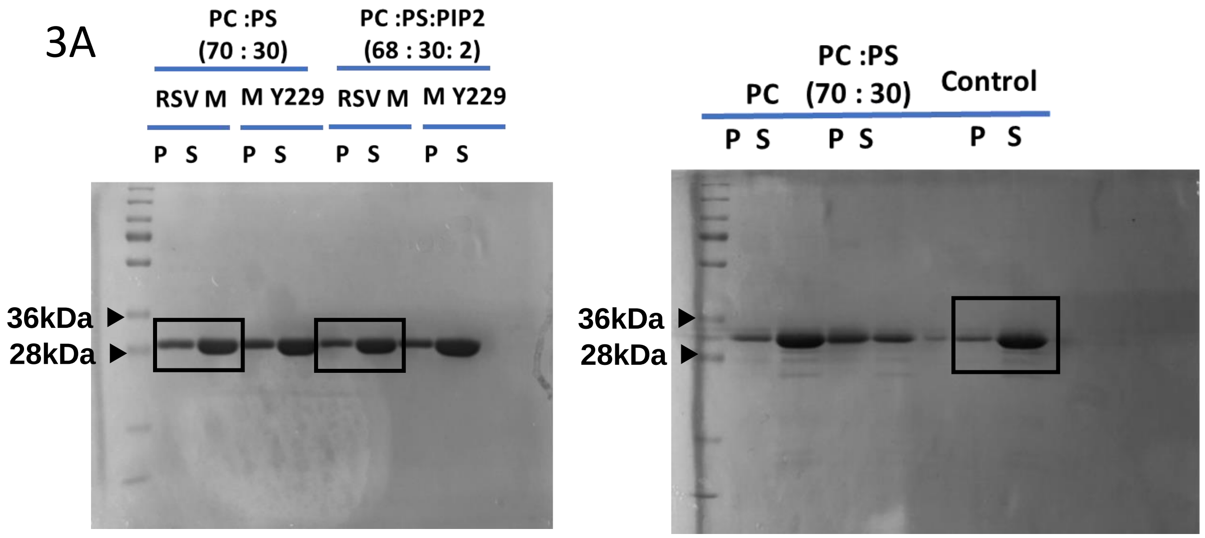
PC :PS
(70 : 30)
PC :PS:PIP2
(68 : 30: 2)
RSV M
M Y229
RSV M
M Y229
P S P S P S P S
36kDa
28kDa
PC
PC :PS
(70 : 30)
Control
P S P S P S
36kDa
28kDa

3B

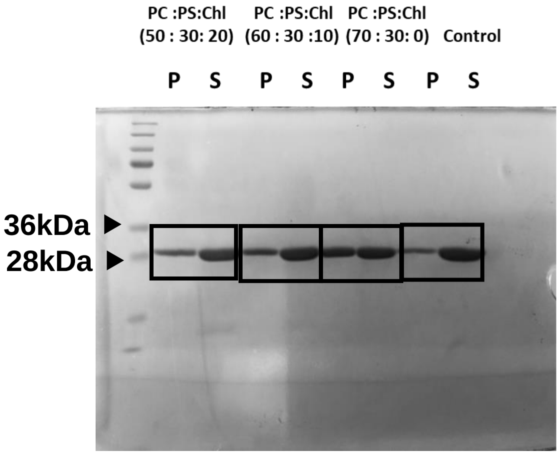
PC :PS:Chl
(50 : 30: 20)
PC :PS:Chl
(60 : 30 :10)
PC :PS:Chl
(70 : 30: 0)
Control
P S P S P S P S
36kDa
28kDa

4B

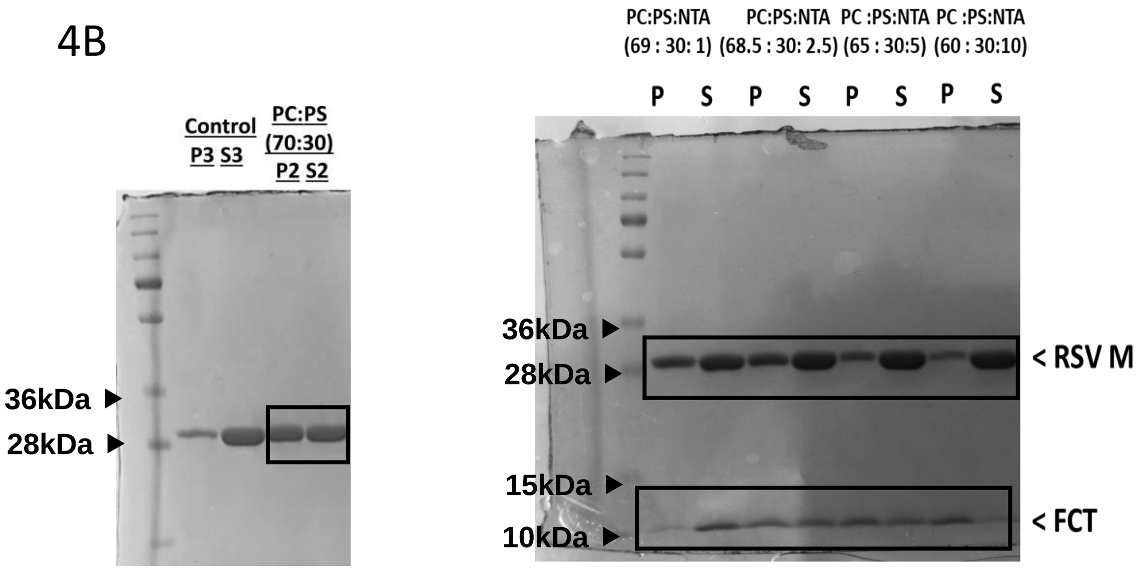
Control
P3 S3
PC:PS
(70:30)
P2 S2
36kDa
28kDa
PC:PS:NTA
(69 : 30: 1)
PC:PS:NTA
(68.5 : 30: 2.5)
PC :PS:NTA
(65 : 30:5)
PC :PS:NTA
(60 : 30:10)
P S P S P S P S
36kDa
28kDa
15kDa
10kDa
< RSV M
< FCT

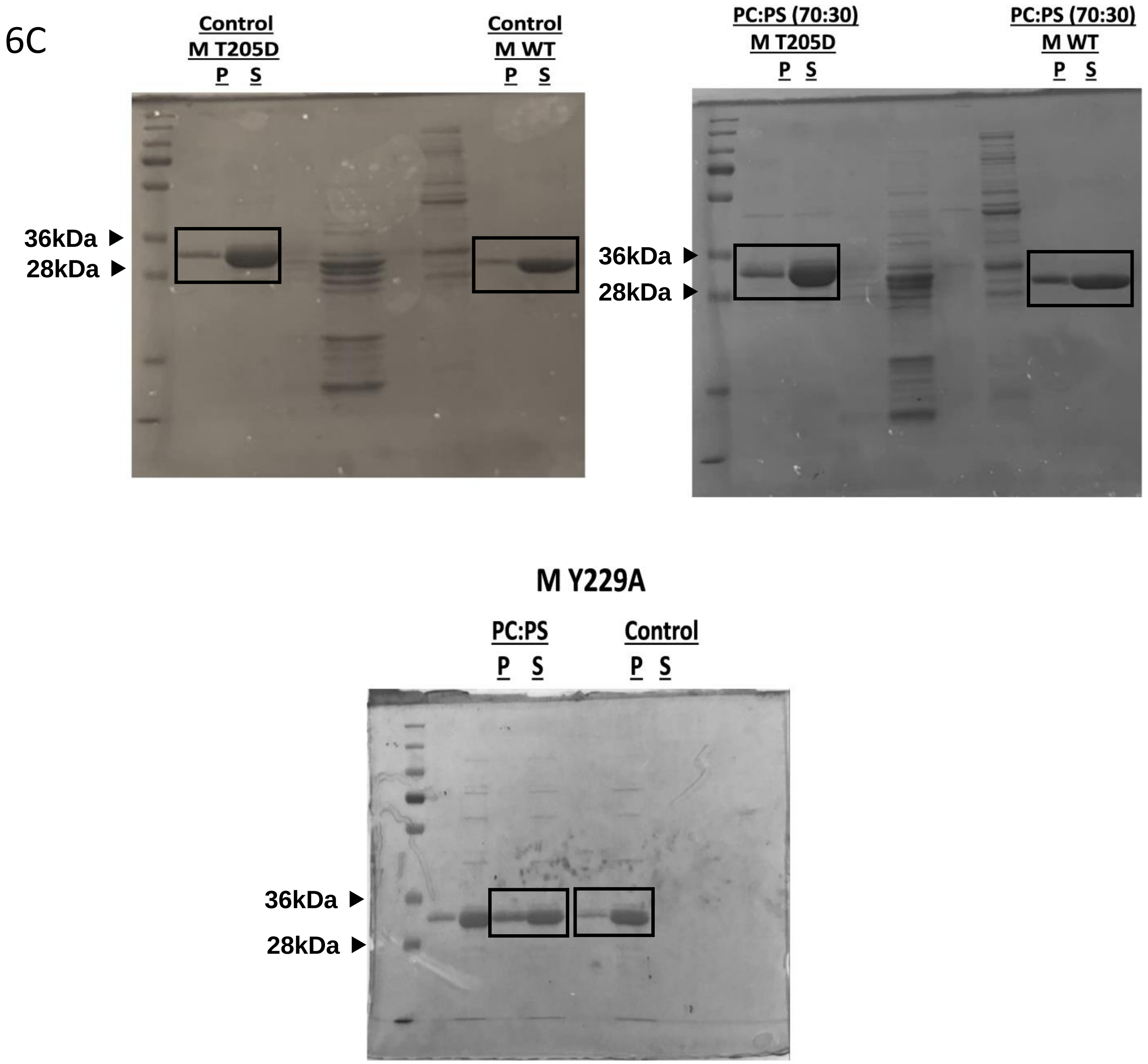

Supplemental Figure 5. Full SDS-PAGE Coomassie gels for 3A, 3B, 4B, and 6C.
The spliced image panels used in the main figures are marked each on its original gel source.

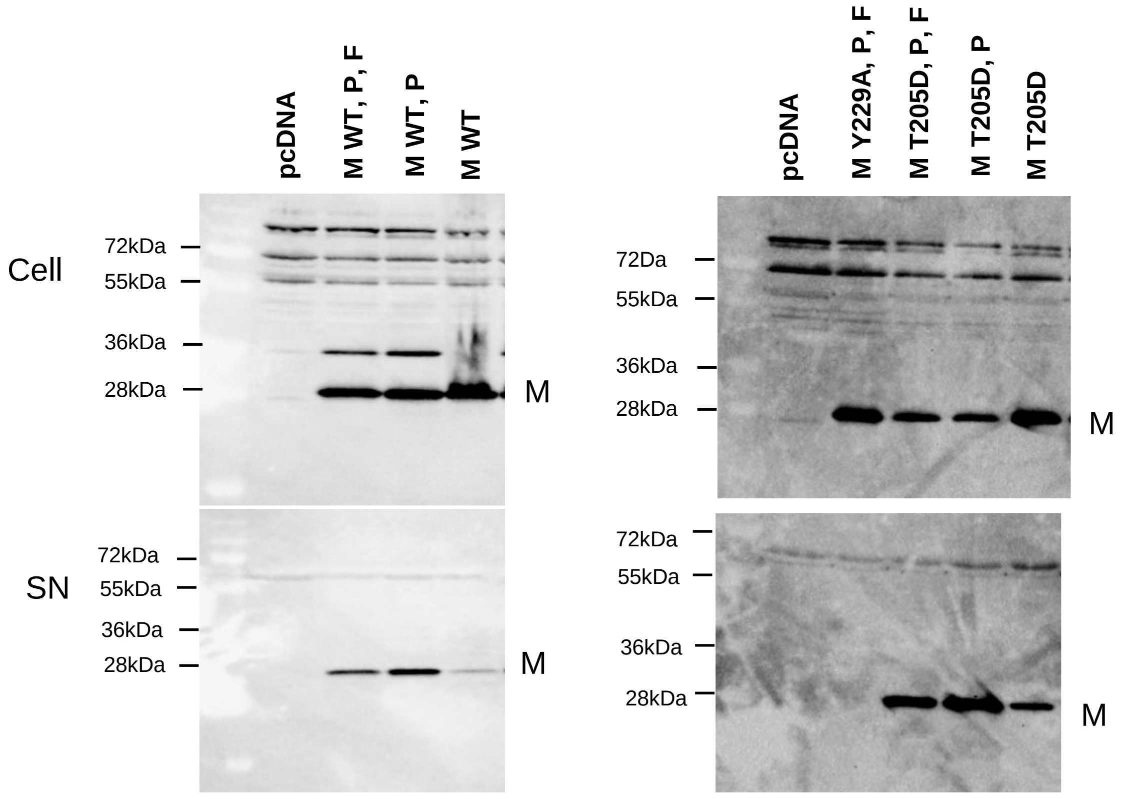

Supplemental Figure 6. Representative full Western Blots for Fig.6B. HEp-2 cells were co-transfected with pcDNA3.1 plasmids expressing RSV M WT, T205D, or Y229A, P and F, or a different combination of these. At 48 h post transfection, cell lysates (top) were generated and VLPs (bottom) were isolated from the supernatant. VLPs and cell lysates were then subjected to Western analysis using anti-M antibody.
